# Analysis of state 1—state 2 transitions by genome editing and complementation reveals a quenching component independent from the formation of PSI-LHCI-LHCII supercomplex in *Arabidopsis thaliana*

**DOI:** 10.1186/s13062-023-00406-5

**Published:** 2023-08-23

**Authors:** Edoardo Andrea Cutolo, Roberto Caferri, Zeno Guardini, Luca Dall’Osto, Roberto Bassi

**Affiliations:** 1 Laboratory of Photosynthesis and Bioenergy, Department of Biotechnology, University of Verona, Strada le Grazie 15, 37134 Verona, Italy; 2 Accademia Nazionale dei Lincei, Palazzo Corsini, Via Della Lungara, 10, 00165 Rome, Italy

**Keywords:** Photosynthesis, Photoacclimation, Reverse genetics, State 1—state 2 transitions, STN7 kinase, Light-harvesting antenna, Site directed mutagenesis, LHCII, Multiplex genome editing

## Abstract

**Background:**

The light-harvesting antennae of photosystem (PS) I and PSII are pigment-protein complexes responsible of the initial steps of sunlight conversion into chemical energy. In natural environments plants are constantly confronted with the variability of the photosynthetically active light spectrum. PSII and PSI operate in series but have different optimal excitation wavelengths. The prompt adjustment of light absorption by photosystems is thus crucial to ensure efficient electron flow needed to sustain downstream carbon fixing reactions. Fast structural rearrangements equilibrate the partition of excitation pressure between PSII and PSI following the enrichment in the red (PSII-favoring) or far-red (PSI-favoring) spectra. Redox imbalances trigger state transitions (ST), a photoacclimation mechanism which involves the reversible phosphorylation/dephosphorylation of light harvesting complex II (LHCII) proteins by the antagonistic activities of the State Transition 7 (STN7) kinase/TAP38 phosphatase enzyme pair. During ST, a mobile PSII antenna pool associates with PSI increasing its absorption cross section. LHCII consists of assorted trimeric assemblies of Lhcb1, Lhcb2 and Lhcb3 protein isoforms (LHCII), several being substrates of STN7. However, the precise roles of Lhcb phosphorylation during ST remain largely elusive.

**Results:**

We inactivated the complete *Lhcb1* and *Lhcb2* gene clades in *Arabidopsis thaliana* and reintroduced either wild type *Lhcb1.3* and *Lhcb2.1* isoforms, respectively, or versions lacking N-terminal phosphorylatable residues proposed to mediate state transitions. While the substitution of Lhcb2.1 Thr-40 prevented the formation of the PSI-LHCI-LHCII complex, replacement of Lhcb1.3 Thr-38 did not affect the formation of this supercomplex, nor did influence the amplitude or kinetics of PSII fluorescence quenching upon state 1—state 2 transition.

**Conclusions:**

Phosphorylation of Lhcb2 Thr-40 by STN7 alone accounts for ≈ 60% of PSII fluorescence quenching during state transitions. Instead, the presence of Thr-38 phosphosite in Lhcb1.3 was not required for the formation of the PSI-LHCI-LHCII supercomplex nor for re-equilibration of the plastoquinone redox state. The Lhcb2 phosphomutant was still capable of ≈ 40% residual fluorescence quenching, implying that a yet uncharacterized, STN7-dependent, component of state transitions, which is unrelated to Lhcb2 Thr-40 phosphorylation and to the formation of the PSI-LHCI-LHCII supercomplex, contributes to the equilibration of the PSI/PSII excitation pressure upon plastoquinone over-reduction.

**Supplementary Information:**

The online version contains supplementary material available at 10.1186/s13062-023-00406-5.

## Introduction

The photosynthetic apparatus is a molecular machinery that catalyzes the conversion of CO_2_ into organic molecules using light energy absorbed by photoexcitable pigments. In the light-dependent photosynthetic reactions, water is oxidized by photosystem II (PSII) and electrons are transported across the thylakoid membrane by the oxidizing activity of PSI to reduce ferredoxin and NADP^+^ [65]. Electron flow is coupled to proton pumping in the thylakoid lumen creating an electrochemical gradient which is dissipated by ATP synthase complex. The ATP and NADPH pools are then re-oxidized in the downstream CO_2_ reduction reactions of the Calvin-Benson cycle and recycled as electron and phospho-group acceptors. PSI and PSII consist of a core complex—the site of initial charge separation which exclusively binds chlorophyll (Chl) a and *β*-carotene—and by a peripheral light-harvesting antenna system. Antennae are composed of arrays of pigment-binding (Chls and xanthophylls) light harvesting complex (Lhc) proteins which ensure efficient photon capture [68] and participate to the acclimation responses to fluctuations of the light environment, including photoprotection against excess irradiance [8]. Since PSI and PSII operate in series, their balanced excitation by incident light must be preserved to avoid the over-oxidation/reduction of components along the electron transport chain [33]. The PSII antenna consists of trimeric Lhcb assemblies (LHCII trimers) [68] and undergoes short-term remarkable remodeling in response to fluctuations of the light environment. PSI and PSII differ in their absorption properties: while PSII is enriched in red light-absorbing chromophores [68], PSI is spectrally shifted towards far-red (FR) wavelengths. Hence, when the incident light is transiently enriched in a specific spectral component, the two PSs are unevenly excited and the overall photosynthetic efficiency decreases. This condition typically occurs within dense canopies, where the uppermost sun-lit foliage absorbs most of the blue and red photons and transmits far-red-enriched light to the lower leaf layers [62]. Under weak irradiance, the excitation balance between PSI or PSII in the shaded foliage is maintained by the short-term acclimation process of state transitions (ST) (John F. [2, 38, 63]. ST are triggered by over-reduction of the plastoquinone pool under PSII-favoring light and activation of the serine-threonine State Transition 7 (STN7) kinase [11]. Upon interaction with cytochrome *b*_*6*_*f* [85] and a redox-dependent activation mechanism in the lumen [104], STN7 dimerizes [105, 106] and phosphorylates stroma-exposed Lhc residues, causing the migration of a subset of the PSII antenna towards the non-appressed lamellae and a transient association of a mobile LHCII trimer pool with PSI to form a PSI-LHCI-LHCII supercomplex [46, 84]. The docking of LHCII to PSI enhances its absorption cross section promoting the oxidation of intermediate electron carriers between PSI and PSII, thereby inactivating the kinase. Under PSI-favoring light, the Thylakoid-associated Phosphatase 38 (TAP38) (Mathias [73, 86] dephosphorylates Lhc proteins reversing their association to PSI. This enables the dynamic equilibration of the electron transport chain redox poise [89]. The antenna fraction shuttling between PSI and PSII consists of LHCII heterotrimers enriched in the Lhcb1 and Lhcb2 proteins [32] which both harbour phosphorylatable residues at their N-termini. Although both Lhcb1 and Lhcb2 are phosphorylated by the STN7 kinase, only P-Lhcb2 is present in the PSI-LHCI-LHCII complex [56], where its phosphorylated N-terminal Thr-40 residue mediates the attachment of the LHCII trimer to the surface of the PSI supercomplex [69] in the thylakoid margins and stroma lamellae, while P-Lhcb1 was enriched in the PSII supercomplexes and mostly present in the inner grana regions [22]. The genetic analysis of isoform-specific Lhcb phosphorylation events, and of their contribution to the process of ST, however, is hampered by the redundancy of the *Lhcb1* and *Lhcb2* gene clades [43]. To dissect the role(s) of individual Lhcb phosphosites, we therefore adopted a multiplex CRISPR-Cas9-based genome editing approach [66] to delete the complete *Lhcb1* or *Lhcb2* gene clades followed by complementation of either *Lhcb1* or *Lhcb2* isoforms lacking the consensus phosphorylatable residues Thr-38 and Thr-40, respectively. These phosphosites were previously designated as targets of STN7 and used for the development of phospho-specific α-Lhcb1/2 antibodies [52]. Here, we demonstrated that the substitution of Lhcb2 Thr-40 (Thr-40) with a non-phosphorylatable valine residue (T→V) locked the plant in state 1, preventing the formation of the PSI-LHCI-LHCII supercomplex and re-equilibration of the plastoquinone redox state. Modification of Lhcb1 Thr-38, instead, had no detectable effect. The Lhcb2 T40V mutation, however, did not fully abolish ST-dependent PSII fluorescence quenching (qT) but reduced its amplitude by ≈ 60%, suggesting that an additional uncharacterized component(s) contributes to the dynamic adjustment of the PSI/PSII excitation balance upon shifts of light quality. We thus provide compelling evidence of the prominent role of Lhcb2 phosphorylation in the remodeling of the PSI/PSII antenna during state transitions and report on the presence of a previously unrecognized, Lhcb2-independent, STN7-dependent component in the redox balance mechanism.

## Results

### Complementation of knockout *Lhcb1/2* genotypes with single gene isoforms restored wild type LHCII levels

**Fig. 1:**
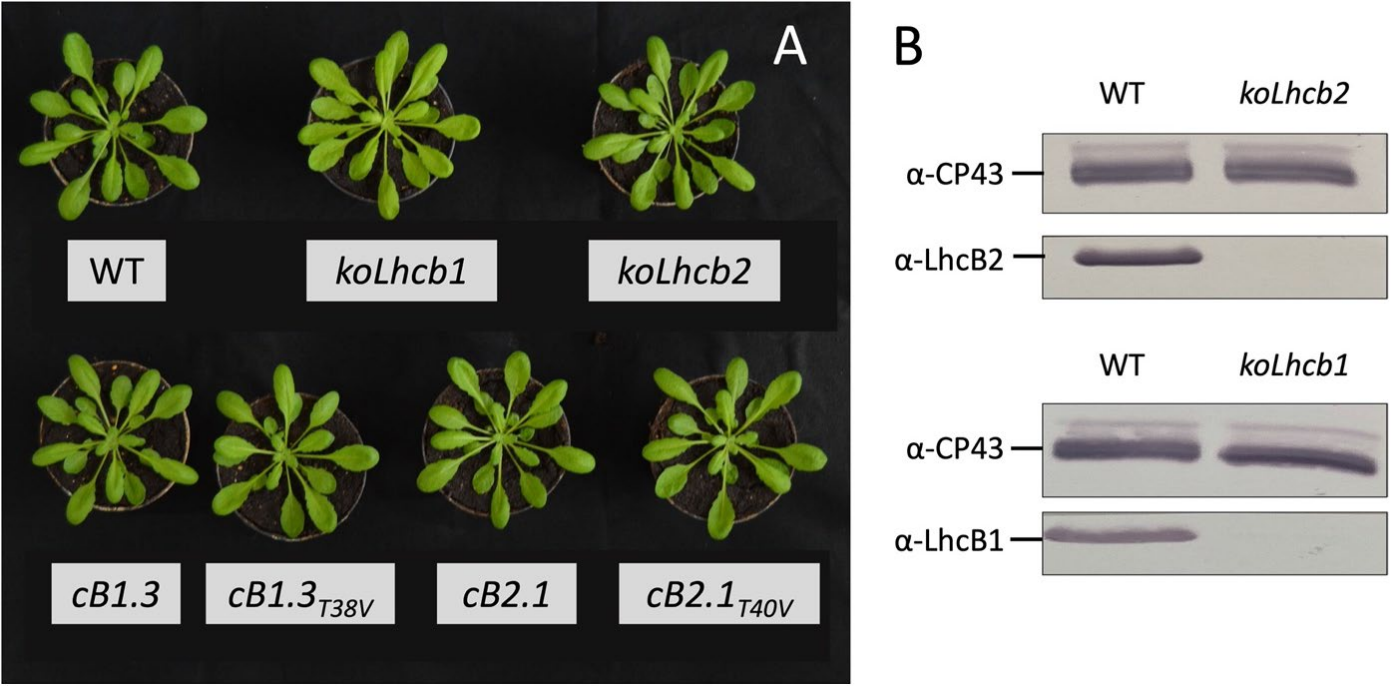
Phenotypes of knockout and complemented genotypes created in this work. **A**: Genotypes used in this study at six weeks after sowing following cultivation under controlled conditions with a 16/8 h night/day regime under 100 μmol photons m^−2^ s^−1^ light intensity (spectrum given in Additional file 1: Fig. S1). The *koLhcb1* mutant exhibited a pale green phenotype while the *koLhcb2* genotype was indistinguishable from the wild type plant (upper row). Neither genotype was affected in their growth. The *cB1.3* and *cB1.3*_*T38V*_ lines resulting from the complementation of the *koLhcb1* genotype recovered the fully green phenotype. **B**: Immunodecoration with α-Lhcb1 α-Lhcb2 antibodies showing the complete absence of Lhcb1 and Lhcb2 proteins in the genome edited *koLhcb*1 and *koLhcb2* background genotypes

To investigate the role(s) of Lhcb1 and Lhcb2 phosphorylation during ST we first developed knockout genotypes impaired in the expression of all Lhcb1 and Lhcb2 protein isoforms. Using two sets of promiscuous gRNAs assembled into multiplex genome editing vectors [66] we targeted highly conserved exonic regions of the *Lhcb1* or *Lhcb2* clades. The resulting *koLhcb1* plants exhibited a pale green phenotype due to the absence of this highly expressed Lhcb protein subfamily (≈ 65% of total Lhcb pool in the wild type) (Stefan [42] as revealed by immunological and biochemical analyses (Fig. 1B and Additional file 1: Fig. S3). This genotype had a higher Chl a/b ratio (4.37 ± 0.15 vs. 3.16 ± 0.04 of wild type, Table 1), consistent with a reduced peripheral antenna [61]. This observation was supported by a drastic reduction in the abundance of LHCII trimers (Additional file 1: Fig. S4) and a lower Chl content per leaf area (11.3 ± 0.8 μg/cm^2^ vs. 17.7 in wild type). The Chl deficiency was associated to a small decrease in F_v_/F_m_ which, however, did not impair growth of *koLhcb1* plants under controlled conditions. The *koLhcb2* genotype, instead, was indistinguishable from the wild type (Fig. 1A), suggesting that the absence of the Lhcb2 protein pool (≈ 25% of total Lhcb pool) (Stefan [42] was compensated by an increased Lhcb1 abundance as previously observed when Lhcb2 synthesis was post-translationally repressed via RNA interference [71](Table 1). All *koLhcb1* genotypes complemented with either native (*cB1.3*) and phospho-mutant (*cB1.3*_*T38V*_) *Lhcb1.3* isoforms recovered wild type levels of LHCII polypeptides and assembled LHCII trimer (Additional file 1: Figs. S3 and S4) leading to the restoration of a fully green phenotype (Fig. 1A) and PSII quantum yield (> 0.81). The immunological characterization of the knockout genotypes confirmed the loss of Lhcb1 or Lhcb2 protein pools and the restoration of wild type levels in the complemented lines (Additional file 1: Fig. 5). Thus, the complementation with single *Lhcb* isoforms successfully reconstituted the levels of Lhcb1 and Lhcb2 protein pools, which are natively encoded by multiple genes [43].

**Table 1:** Pigment composition and F_v_/F_m_ values of all genotypes employed in this work

Genotype	Chl a/b	Chl/Car	F_v_/F_m_
WT	3.16 ± 0.04	3.87 ± 0.12	0.81 ± 0.009
*kostn7*	2.98 ± 0.03	4.19 ± 0.09	0.81 ± 0.005
*koLhcb1*	4.37 ± 0.15	4.24 ± 0.17	0.79 ± 0.012
*koLhcb2*	3.12 ± 0.05	3.96 ± 0.22	0.82 ± 0.009
*cB1.3* #1	3.05 ± 0.04	4.24 ± 0.14	0.81 ± 0.007
*cB1.3* #2	2.99 ± 0.01	4.25 ± 0.13	0.81 ± 0.003
*cB1.3*_*T38V*_ #1	2.87 ± 0.06	3.94 ± 0.27	0.81 ± 0.013
*cB1.3*_*T38V*_ #2	2.98 ± 0.05	4.10 ± 0.27	0.81 ± 0.004
*cB2.1* #1	3.20 ± 0.08	3.99 ± 0.15	0.81 ± 0.01
*cB2.1* #2	3.13 ± 0.08	4.11 ± 0.34	0.81 ± 0.005
*cB2.1*_*T40V*_ #1	3.09 ± 0.06	4.34 ± 0.09	0.81 ± 0.004
*cB2.1*_*T40V*_ #2	2.90 ± 0.05	4.02 ± 0.12	0.83 ± 0.007

### Lhcb1 Thr-48 and Lhcb2 Thr-40 are genuine STN7-dependent phosphosites

**Fig. 2:**
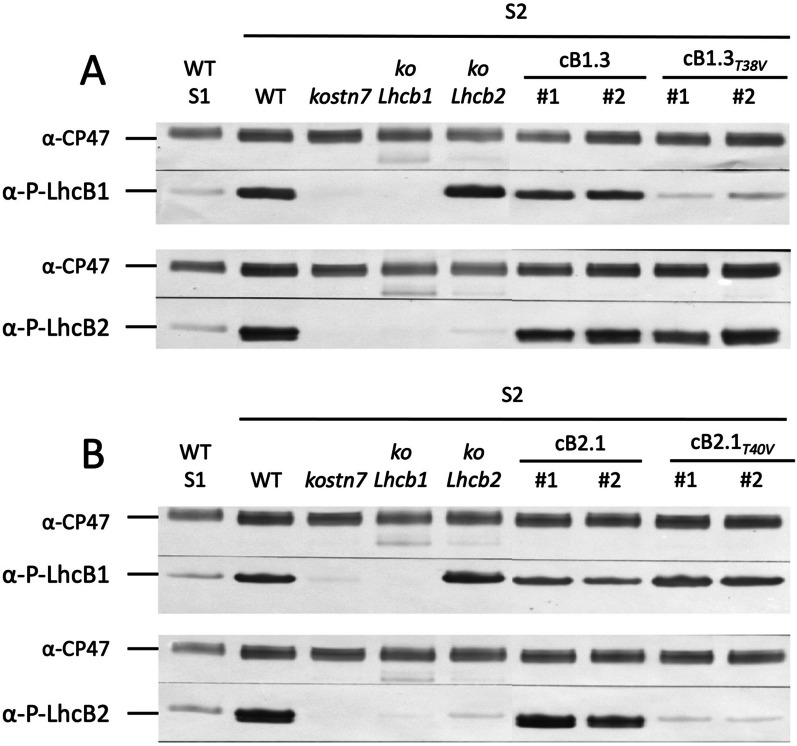
Immunodecoration of phosphorylated Lhcb1 and Lhcb2 epitopes. The wild type positive control (adapted to state 2, PSII-favoring light) and two negative controls (dark-adapted wild type, equivalent to state 1, and *kostn7* mutant) were immunologically probed with α-P-Lhcb1 α-P-Lhcb2 antisera. Panel** A**: in vivo phosphorylation status of Lhcb1 Thr-38 in the *koLhcb1* and *Lhcb2* background genotypes and two independent complemented lines carrying the wild type Lhcb1.3 gene copy or the phosphomutant version lacking the phosphorylatable residue (*cB1.3* and *cB1.3*_*T38V*_) Panel** B**: the in vivo phosphorylation status of Lhcb2 Thr-40 in the *koLhcb1* and *Lhcb2* background genotypes and in two independent complemented lines (*cB2.1* and *cB2.1*_*T40V*_). Approximately 0.5 μg of Chl were loaded for each sample

The relevance of selected Lhcb phosphosites was investigated immunologically using antisera specific for phosphorylated Lhcb1 and Lhcb2 epitopes at position Thr-38 and Thr-40, respectively [22, 52, 71]. Following treatment with PSII-favoring light, strong signals corresponding to phosphorylated Lhcb1 and Lhcb2 pools could be detected in the wild type and in two independent lines complemented with wild type *Lhcb1.3* and *Lhcb2.1* versions (*cB1.3* and *cB2.1*), respectively. As expected, in the *kostn7* genotype lacking the LHCII kinase, no reactive bands could be observed in any tested condition. Similarly, no phosphorylation could be detected in the lines complemented with the phosphomutant *Lhcb1.3*_*T38V*_ and *Lhcb2.1*_*T40V*_ versions (*cB1.3*_*T38V*_ and *cB2.1*_*T40V*_) (Fig. 2A and B). However, a slight cross-reactivity of the α-P-Lhcb2 antiserum against, presumably, phosphorylated versions of Lhcb1 proteins was observed, as this faint signal was entirely missing in the *koLhcb1* and *kostn7* samples. The α-P-Lhcb1 antiserum, instead, revealed a low reactivity in the light-adapted *cB1.3*_*T38V*_ lines. This observation is tentatively explained by a mild cross-reactivity of the antiserum against the endogenous P-Lhcb2 pool since this reaction is absent in ko*stn7* and *kolhcb1*. Finally, in the dark-adapted wild type (state 1) we detected faint α-P-Lhcb1/2 reactive bands. This observation can be tentatively explained by the metabolic control of STN7 activity upon plastoquinone reduction by the products of starch degradation [41]. Our results confirmed that Lhcb1 Thr-38 and Lhcb2 Thr-40 are *bona fide* substrates of the STN7 kinase and that their phosphorylation is predominantly light-dependent, although not exclusively. Moreover, the *koLhcb1* line treated with PSII-favouring light failed to phosphorylate the endogenous Lhcb2 pool, in agreement with a previous report [71, 79] which suggested that the smaller LHCII antenna of this genotype prevents the redox-dependent activation of the STN7 kinase at the light intensity used to induce ST [85]. A stronger phosphorylation of the endogenous Lhcb1 pool was observed in the *koLhcb2* and *cB2.1*_*T40V*_ genotypes as compared with the wild type and the *cB2.1* line. We suggest this is caused by the enhanced activity of the STN7 due to a sustained plastoquinone reduced state in the genotypes impaired in ST.

### STN7-dependent Lhcb2 Thr-40 phosphorylation mediates the formation of the PSI-LHCI-LHCII supercomplex

**Fig. 3:**
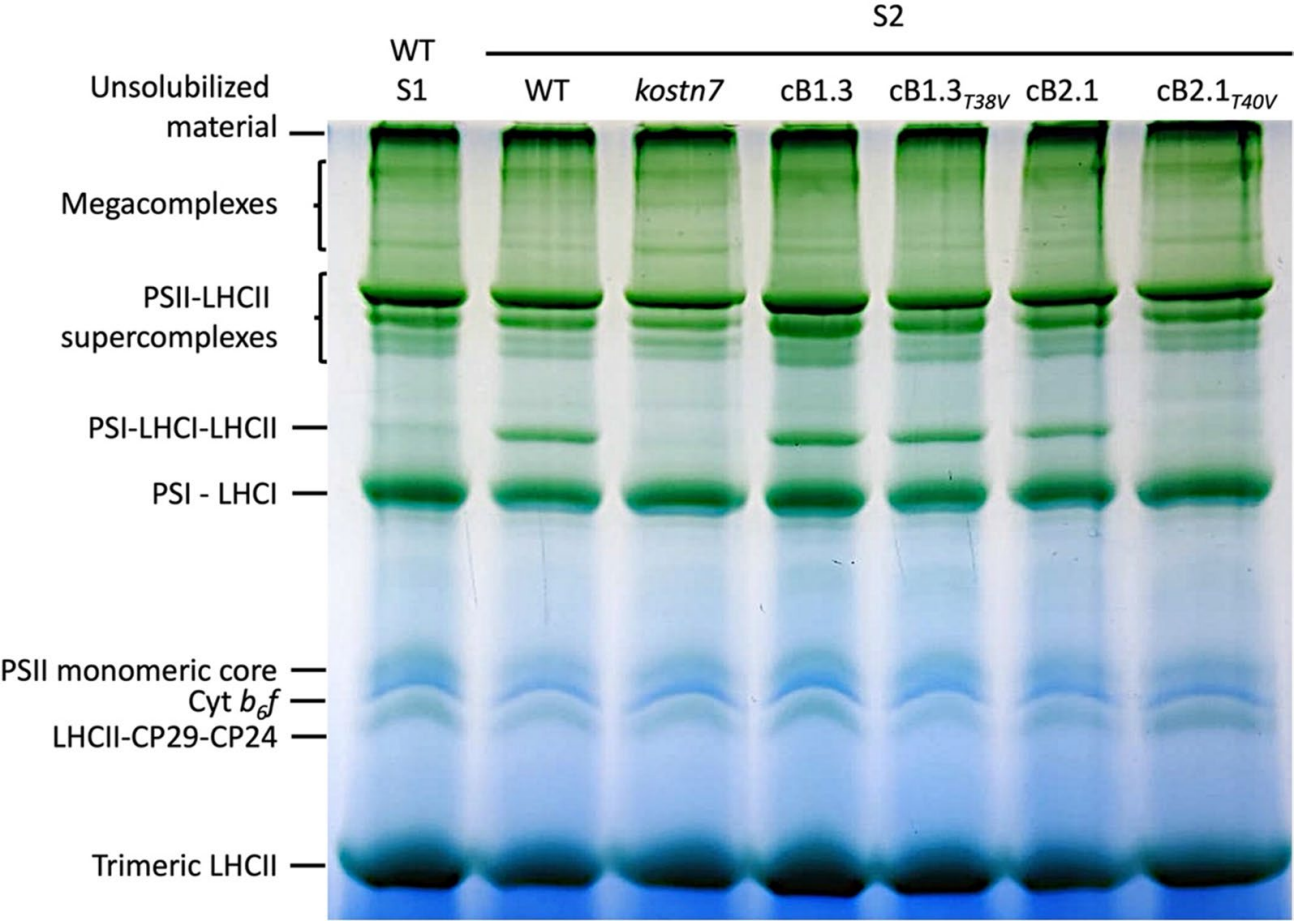
Identification of the PSI-LHCI-LHCII supercomplex induced by PSII-favouring light. The contribution of Lhcb1 and Lhcb2 phosphorylation to the establishment of the PSI-LHCI-LHCII supercomplex was investigated following treatment with PSII-favoring light for 2 h. Isolated thylakoids (35 μg of Chl) were solubilized with 1% (w/v) digitonin and intact protein complexes separated using a non-denaturing a Large Pore Blue Native (lpBN) gel. A dark-adapted (equivalent to state 1) and the *kostn7* mutant were employed as negative controls. The PSI-LHCI-LHCII supercomplex was observed in all complemented lines, except for the *cB2.1*_*T40V*_ genotype lacking Thr-40 residues, revealing the crucial role of its STN7-dependent phosphorylation in the formation of this complex

The requirement for Lhcb1/2 phosphorylation to induce the formation of a PSI-LHCI-LHCII supercomplex during state 1—state 2 transition was investigated biochemically using a non-denaturing (lpBN) gel system [44]. To this end, samples from dark-adapted wild type (corresponding to state 1) or the *kostn7* mutant were employed as negative controls to verify the formation of a high-molecular weight green band induced by treatment with PSII-favouring light (William H. J. [103]. The formation of the PSI-LHCI-LHCII supercomplex was evident in the wild type as well as in the *cB1.3, cB2.1* lines (Fig. 3) and, notably, in the *cB1.3*_*T38V*_ line in which the Lhcb1 Thr-38 residue targeted by STN7 was absent. In contrast, removal of Lhcb2 Thr-40 prevented the assembly of the supercomplex in the *cB2.1*_*T40V*_ line, indicating that phosphorylation of this residue by STN7 was necessary to promote the stable connection of the mobile LHCII trimers to PSI.

### The phosphorylated Lhcb2 protein pool is enriched in the PSI-LHCI-LHCII supercomplex

**Fig. 4:**
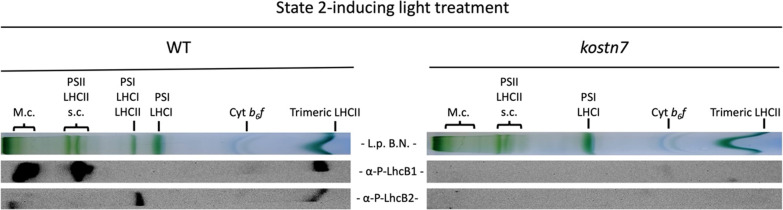
Immunological analysis of the localization of Lhcb1 and Lbcb2 phosphoisoforms in the thylakoid complexes resolved via non-denaturing lpBN gel. Lanes corresponding to state 2-adapted wild type and *kostn7* genotypes were excised from the native gel, denatured, and blotted. Immunodecoration was performed with α-P-Lhcb antisera recognizing phosphorylated epitopes carrying P-Thr-38 and P-Thr-40 of Lhcb1 and Lhcb2, respectively. The P-Lhcb1 pool was exclusively found in the high molecular weight PSII-LHCII supercomplexes and megacomplexes while the P-Lhcb2 pools was enriched in the band corresponding to the PSI-LHCI-LHCII supercomplex

The protein supercomplexes resolved via lpBN gel were subsequently probed immunologically to investigate the localization of phosphorylated Lhcb isoforms. To this aim, lanes from the lpBN gel were blotted and probed with anti P-Lhcb1 and P-Lhcb2 antibodies. We observed that the PSI-LHCI-LHCII supercomplex of the wild type plant contained P-Lhcb2 but no traces of P-Lhcb1 (Fig. 4, left panel). P-Lhcb2 was also detectable in the trimeric LHCII band and in the very high molecular weight band close to the interface with the stacking gel. P-Lhcb1, instead, was enriched in the PSII-LHCII supercomplexes, megacomplexes and, to a lower extent, in the band corresponding to detached trimeric LHCII. No signal for either antibody could be detected in the supercomplexes of the PSII light-treated *kostn7* mutant (Fig. 4, right panel), confirming the strict STN7-dependent phosphorylation of Lhcb1 and Lhcb2, in agreement with previous work [22, 56].

### Phosphorylation of Lhcb2 Thr-40, not Lhcb1 Thr-38, mediates PSII fluorescence quenching and redox equilibration of the plastoquinone pool during ST

**Fig. 5:**
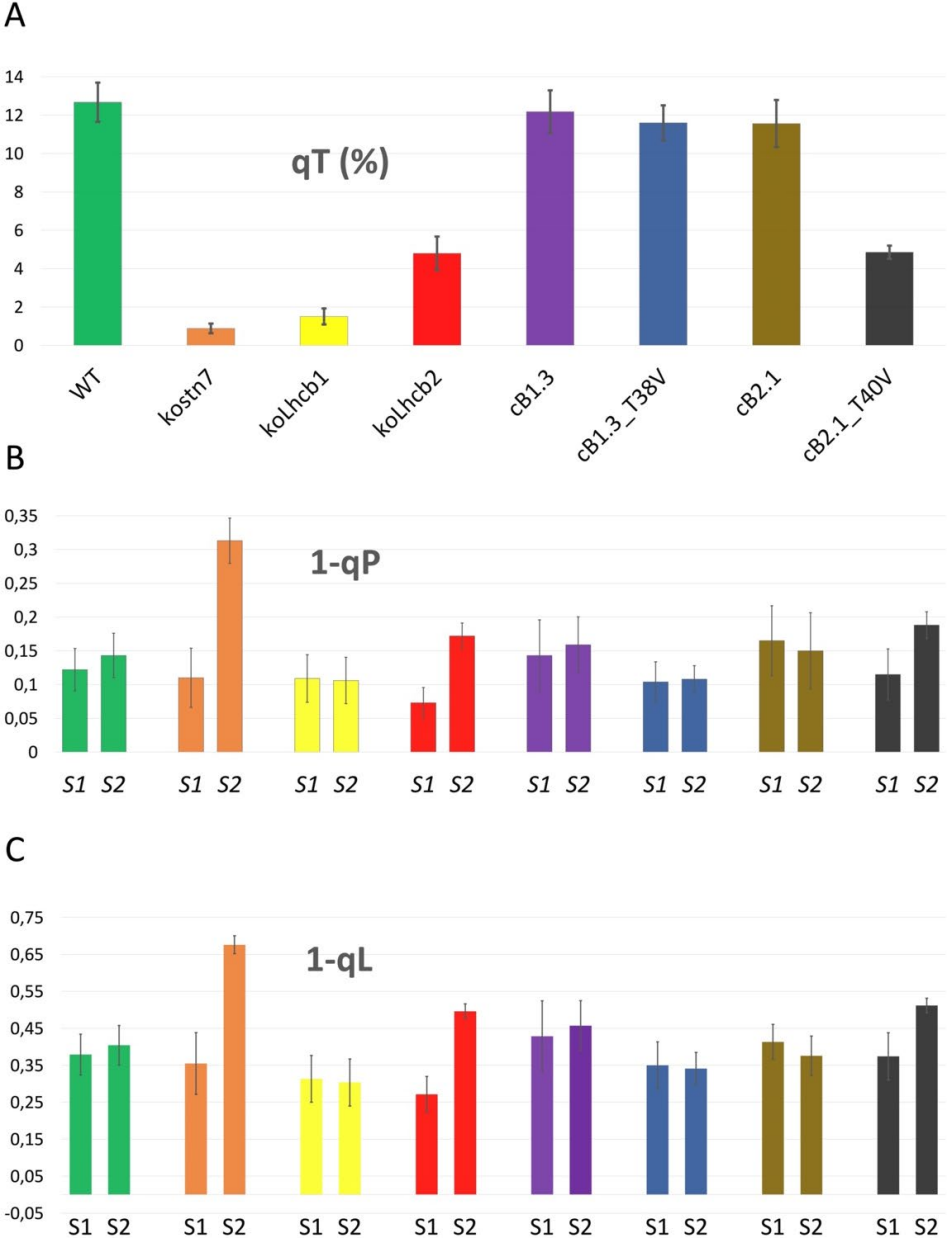
Analysis of PSII fluorescence quenching upon state 1—state 2 transition. The amplitude of PSII fluorescence quenching upon state 1–2 transition was estimated by calculating the qT parameter. **A**: qT values of genotypes. Only the *cB2.1*_*T40V*_, *koLhcb2* and *kostn7* exhibited a reduced qT compared to the wild type. **B** and **C**: 1-qP and 1-qL parameters, which reflect the fraction of closed PSII reaction centers, thus the reduction level of the plastoquinone pool. No change in 1-qP (1-qL) between state 1 and state 2 (S1, S2) indicates a full capacity to equilibrate the PSII/PSI excitation pressure upon shifts in light quality. The *cB2.1*_*T40V*_ genotype, which is impaired in ST-induced PSII fluorescence quenching, failed to equilibrate the plastoquinone redox status

**Fig. 6:**
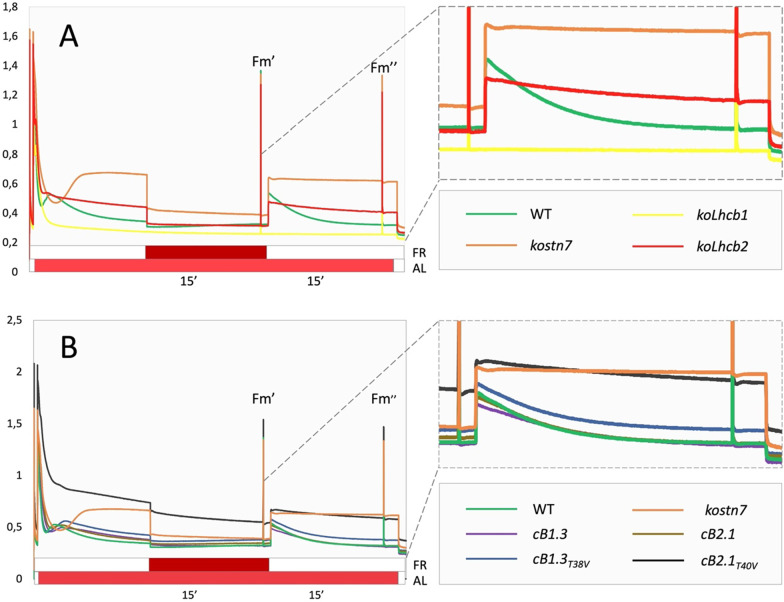
PAM fluorescence traces recorded during state 1—state 2 transition measurement protocol. The PSII fluorescence quenching kinetics upon state 1–2 transition were derived from the PAM recording used to estimate the qT, 1-qP and 1-qL parameters (Fig. 5). The superimposition of far-red (FR) light to a basal actinic light source (AL) was employed to induce the state 1, while removal of the former induced a state 2 condition. All genotypes with reduced qT values (Fig. 5) also exhibited altered fluorescence trace profiles. The *kostn7* mutant displayed the most extreme fluorescence phenotype, while the *koLhcb2* and *cB2.1*_*T40V*_ both displayed a sustained fluorescence trace profile during state 1—state 2 transition, consistent with their inability to assemble the PSI-LHCI-LHCII supercomplex, reduce the PSII antenna absorption cross section and relieve the over-reduction of the plastoquinone pool

All genotypes were investigated by pulse amplitude-modulated (PAM) Chl fluorescence analysis to determine the parameter qT which estimates the amplitude of photochemical quenching of PSII Chl fluorescence by PSI [59]. All genotypes, except for the *kostn7* mutant, exhibited far-red light-induced fluorescence decline, although with different kinetics and amplitude. A maximal qT amplitude was observed in the wild type (qT = 12.6%) and very similar values in the *cB1.3, cB1.3*_*T38V*_ and *cB2.1* genotypes, while the *cB2.1*_*T40V*_ line lacking Lhcb2 Thr-40 exhibited a significantly lower qT (4.85 vs. 12.6; 38.5% of wild type) (Fig. 5A and Table 2). The absence of Lhcb1 Thr-38, instead, did not impair ST-induced PSII fluorescence quenching, consistently with the unaffected assembly of the PSI-LHCI-LHCII supercomplex (Fig. 3). Notably, the *cB2.1*_*T40V*_ line exhibited a similar qT of the parental genotype *koLhcb2*, emphasizing the prominent role of Lhcb2 phosphorylation in ST*.* A drastically dampened qT score was also observed in the *koLhcb1* genotype (1.5 vs. 12.6; ≈ 15% of wild type). In agreement with a previous report [71] (Additional file 1: Figs. S2 and S3), we attribute this effect to the smaller antenna of this genotype which under weak actinic light (AL) fails to reach the plastoquinone reduction threshold required to activate the STN7 kinase. Moreover, this explains the lack phosphorylation of the endogenous Lhcb2 pool (Fig. 2B) and the absence of the transient fluorescence rise in the dark-adapted plant at the onset of the actinic light treatment (Additional file 1: Fig. S7). Upon complementation with Lhcb1_T38V_ neither the PSI-LHCI-LHCII supercomplex, nor the fluorescence decay were affected, despite the fully reconstituted antenna size and the high PQ reduction state (Figs. 2a and 5). The efficiency of the different genotypes to equilibrate the redox poise of the electron transport chain was estimated by the parameter 1-qP calculated at the end of state 1 and state 2 intervals. 1-qP is calculated from the photochemical quenching (qP) parameter and reflects the relative extent of reduction of the primary electron acceptor of PSII Q_A_ [83]. In the wild type plant, 1-qP remains relatively stable upon the shift from PSI- to PSII-favouring light thanks to the activation of ST (Fig. 5B and Table 2). The analogous parameter 1-qL (Fig. 5C and Table 2), instead, reflects the fraction of closed PSII reaction centres assuming a functional connection among PSII units via shared antennae (lake model, L) [51]. While the wild type displayed marginal differences for 1-qP and 1-qL between S1 and S2 states, implying efficient re-equilibration of the redox state of inter-system electron carriers, the *kostn7* mutant exhibited the strongest strong redox unbalance (Δ 1-qP = 0.2; Δ 1-qL = 0.32), implying that the redox re-equilibration was impaired consistent with the null qT. Efficient redox re-equilibration was observed in all complemented lines possessing the Lhcb2 Thr-40 residue. The *koLhcb2* and *cB2.1*_*T40V*_ genotypes, instead, exhibited an altered Q_A_ reduction state following treatment with PSII-favouring light as evidenced by qT, Δ 1-qP, and Δ 1-qL values intermediate between the wild type and the *kostn7* mutant. We also noticed that the presence of the phosphorylatable Lhcb1 Thr-38 was irrelevant for the plant to perform ST. Despite its low qT, the *koLhcb1* genotype exhibited similar 1-qP and 1-qL values between state 1 and state 2, suggesting that the redox equilibrium of the plastoquinone pool was not perturbed by selective PSII excitation, likely because of the drastically reduced size of PSII antenna. The efficiency of redox equilibration upon state 1—state 2 transition could also be deduced from the recorded fluorescence traces (Fig. 6). Except for the *koLhcb1* line (yellow trace in Fig. 6A), which maintained a flat trace irrespective of the light treatment, all other genotypes with reduced qT had trace profiles which significantly deviated from that of the wild type (green trace in Fig. 6A). In particular, the *kostn7*, *koLhcb2* (orange and red traces, respectively) and *cB2.1*_*T40V*_ (black trace) genotypes exhibited disrupted quenching kinetics upon transition from state 1 (AL + FR) to state 2 interval (AL alone). In the wild type, and in the *cB1.3, cB1.3*_*T38V*_ and *cB2.1* lines, the abrupt fluorescence increase caused by the sudden removal of the FR light was followed by a steady decline and return to basal levels. In contrast, in the ST-impaired genotypes (orange *kostn7*; red *koLhcb2*; black *cB2.1*_*T40V*_ traces, respectively) the fluorescence level upon removal of FR light was either maintained constant (orange) during the following 15 min, or declined very slowly (red, and black) owing to the inability of the apparatus to reversibly acclimate to light quality shifts and photochemically quench the enhanced PSII Chl fluorescence upon removal of the far-red light.

**Table 2:** Summary of qT, 1-qP and 1-qL in state 1 and state 2 for all genotypes

Genotype	Wild type	*kostn7*	*koLhcb1*	*koLhcb2*	*cB1.3*	*cB1.3*_*T38V*_	*cB2.1*	*cB2.1*_*T40V*_
qT	12.6 ± 1.02	0.88 ± 0.24	1.5 ± 0.41	4.8 ± 0.86	12.17 ± 1.1	11.6 ± 0.9	11.5 ± 1.2	4.85 ± 0.34
% of WT qT	–	6.98	11.9	38	96.6	92	91.6	38.5
1-qP (S1)	0.12 ± 0.03	0.11 ± 0.04	0.11 ± 0.07	0.07 ± 0.02	0.14 ± 0.05	0.1 ± 0.03	0.16 ± 0.09	0.11 ± 0.03
1-qP (S2)	0.14 ± 0.03	0.31 ± 0.03	0.1 ± 0.07	0.17 ± 0.02	0.16 ± 0.04	0.1 ± 0.02	0.15 ± 0.08	0.18 ± 0.02
Δ 1-qP (S2-S1)	0.023	0.203	− 0.003	0.099	0.016	0.004	− 0.015	0.073
1-qL (S1)	0.38 ± 0.05	0.35 ± 0,08	0.31 ± 0.16	0.27 ± 0.05	0.43 ± 0.095	0.35 ± 0.06	0.41 ± 0.14	0.37 ± 0.06
1-qL (S2)	0.4 ± 0.05	0.67 ± 0.02	0.3 ± 0.16	0.49 ± 0.02	0.45 ± 0.067	0.34 ± 0.043	0.35 ± 0.15	0.51 ± 0.02
Δ 1-qL (S2-S1)	0.021	0.321	− 0.01	0.225	0.029	− 0.009	− 0.056	0.137

### Lhcb serine phosphorylation is STN7- and light-independent

**Fig. 7:**
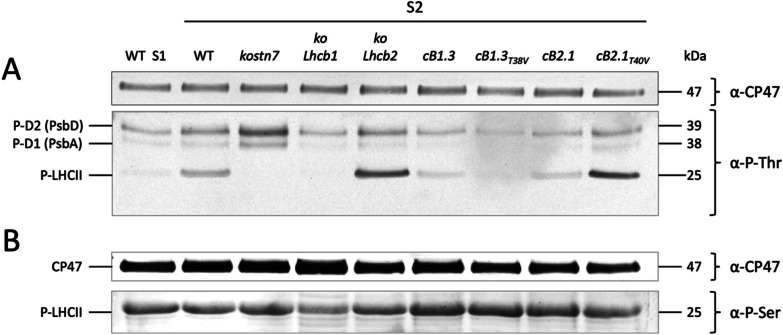
Immunodecoration of thylakoid phosphoproteins with α-P-Thr and α-P-Ser antibodies. Isolated thylakoid samples corresponding to approximately 0.5 and 5 μg of Chl were blotted following separation on SDS PAGE and probed with α-P-Thr (panel** A**) and α-P-Ser antibodies (panel** B**), respectively. Experiments included all genotypes created in this work treated with PSII-favoring light and a dark-adapted (equivalent to state 1) wild type sample control. The pattern of the α-P-Thr reaction (panel** A**) revealed the characteristic thylakoid phosphoproteins D1 (PsbA), D2 (PsbD) and LHCII. Consistent with the results presented in figure 2, the LHCII signal was extremely low in the dark-adapted wild type and was entirely missing in the *koLhcb1* and *kostn7* genotypes. *kostn7* (and to lower extent the *koLhcb2* and *cB2.1*_*T40V*_ lines) exhibited a stronger Thr phosphorylation of the PSII core complex subunits D1 and D2. Enhanced LHCII Thr phosphorylation was observed in the *koLhcb2* and *cB.1*_*T40V*_ lines because of persistent plastoquinone reduction and the active state of the STN7 kinase towards the Lhcb1 Thr-38 residue. The α-P-Ser reaction (panel** B**), instead, revealed an equal phosphorylation level of the LHCII band in all genotypes, except for the *koLhcb1* sample where the faint reactive band corresponds to the phosphorylated serine(s) belonging to Lhcb2 polypeptides

The results presented in the previous sections indicate a strong correlation between the ability to redox-equilibrate the plastoquinone pool (Fig. 5) and the Lhcb2 Thr-40 phosphorylation-dependent fluorescence decay (Fig. 6). However, Thr-40 mutation, in the *cB2.1*_*T40V*_ line, did not fully suppress qT activity, nor reproduced the maximal plastoquinone redox unbalance observed in the *kostn7* mutant (see Fig. 5 and Δ 1-qP and Δ 1-qL values in Table 2). Thus, beside Lhcb2 Thr-40 phosphorylation, additional STN7-dependent process(es) contributed to the above-described regulation in *koLhcb2* and *cB2.1*_*T40V*_ lines and accounted for the residual ≈ 40% fluorescence quenching (Figs. 5A and 6B). Thus, we proceeded to verify whether, in addition to Lhcb2 Thr-40, other Lhcb1 phosphorylation events could contribute to STN7-mediated activation of ST. To this end, we probed total thylakoid protein extracts with α-phosphothreonine (α-P-Thr) and α-phosphoserine (α-P-Ser) antibodies targeting polypeptides bearing P-Thr and P-Ser residues irrespective from the sequence context. The α-P-Thr reaction highlighted two bands of 38 and 39 kDa matching, respectively, the PSII core D1 (PsbA) and D2 (PsbD) subunits [14], and a lower band of 25 kDa corresponding to the unresolved Lhcb1 and Lhcb2 polypeptides. In agreement with the experiments performed using the epitope-specific α-P-Lhcb1/2 antibodies (Fig. 2A and B), we observed that Lhcb threonine phosphorylation required the exposure to PSII-favoring light and was strictly STN7-dependent (Fig. 7A), since only a faint signal appeared in the dark-adapted (state 1) wild type sample while no band at all was detectable in the *kostn7* mutant. Again, the smaller PSII antenna of the *koLhcb1* genotype (Additional file 1: Fig. S2) prevented light-induced threonine phosphorylation in Lhcb2. The signal intensity of the Lhcb-specific α-P-Thr reaction varied significantly between complemented lines with the strongest band detected in the *koLhcb2* and *cB2.1*_*T40V*_ genotypes, while the *cB1.3* and *cB2.1* lines exhibited wild type levels. This observation is consistent with the α-P-Lhcb1/2 reactions presented in Figs. 2A and B and reflects the enhanced phosphorylation of Lhcb1 Thr-38 by STN7 when the key Lhcb2 Thr-40 residue is replaced by valine. The very faint α-P-Thr reactive band in the *cB1.3*_*T38V*_ line, instead, corresponded exclusively to the less-abundant P-Lhcb2 pool owing to the absence of Thr-38 in the dominant Lhcb1 isoform(s). The α-P-Ser reaction had a contrasting pattern, with a similar signal intensity in all genotypes irrespective of the light treatment and the presence of the STN7 kinase (Fig. 7B). We also observed a stronger Thr phosphorylation of the PSII core subunits D1 and D2 in the *kostn7*, *koLhcb2* and *cB2.1*_*T40V*_ which are, to different extents, impaired in ST-mediated fluorescence quenching (qT) and unable to re-equilibrate the plastoquinone redox state (Fig. 5) upon state 1—state 2 transition, an effect attributable to the enhanced activity of the paralog kinase STN8 [105, 106].Taken together, these results suggest that Lhcb1 Thr-38 and Lhcb2 Thr-40 are the main light-dependent LHCII targets of STN7, whereas the phosphorylation of Lhcb1 and Lhcb2 serine residues required the light-independent activity of an uncharacterized kinase(s) and, thus, is not related to ST.

## Discussion

### Genetic deconstruction of Lhcb phosphosites

To better understand the contribution of the phosphorylation of individual Lhcb isoforms to the process of ST, we employed a reverse genetics approach coupled to functional complementation. Initially we obtained the complete inactivation of the *Lhcb1* and *Lhcb2* gene clades and then reintroduced either wild type *Lhcb1*/*2* sequences or mutant versions lacking single consensus phosphorylatable residues. We selected Lhcb1.3 P-Thr-38 and Lhcb2.1 P-Thr-40 (41 in Lhcb2.3) owing to their unambiguous assignment within the tryptic peptides (pT)VAKPKGPSGSPWYGSDRVK and (pT)VKSTPQSIWYGPDRPK [93], respectively, and because they have been experimentally reported in independent proteomic analyses [30, 78, 95, 107]. The mobile LHCII trimer population that transiently associates with PSI during ST consists of heterogenous assemblies of Lhcb1 and Lhcb2 isoforms with a negligible contribution of Lhcb3 [9]. This “extra” trimer population is also referred to as loosely bound (L), owing to its weak association to the PSII core complex [32]. While Lhcb1 and Lhcb2 protein families share similar pigment composition and include phosphoisoforms, the single gene product Lhcb3 is spectroscopically distinct [17]. Although Lhcb3 appears to influence the rate of ST of in *A thaliana* [27], the mature protein was not reported to carry phosphosites [43]; thus, *Lhcb3* was excluded from our gene editing scheme. Importantly, the three *Lhcb2* genes encode identical mature proteins, while Lhcb1.4 and Lhcb1.5 differ from the Lhcb1.1–3 isoforms having six and three amino acid positions substitutions, respectively, which, in the case of Lhcb1.4 include the phosphorylatable Thr-38 residue targeted by STN7. Therefore, we employed the highly expressed *Lhcb1.3* and *Lhcb2.1* gene isoforms [43] to restore wild type levels of Lhcb1 and Lhcb2 protein pools in the *koLhcb1* the *koLhcb2* genotypes, respectively. By doing so, we achieved full complementation of Lhcb1 and Lhcb2 pools (Additional file 1: Fig. S4) and re-established wild type LHCII trimer levels (Additional file 1: Fig. S3). Our work also demonstrated that complementation with single Lhcb sequences—notably of the Lhcb1.3 member of the diversified Lhcb1 sub-family—successfully reconstituted a physiological mechanism to which contribute multiple isoforms in wild type plants. Reduced levels of the STN7 kinase were detected in the *cB2.1* (≈ 25% less than wild type) and, to a greater extent, in the *cB2.1*_*T40V*_ genotype (≈ 50% of wild type) (Additional file 1: Fig. S6). A similar effect was reported in a previous work in which autophosphorylation sites of STN7 were mutated [101] and yet LHCII phosphorylation levels were unaffected, implying that the kinase abundance is not limiting in the process. In support of this view, the estimated Lhcb1/STN7 stoichiometric ratio in *A. thaliana* was 595/1 [60], suggesting an extremely fast catalytic activity of the kinase towards Lhcb substrates. We also observed that lower STN7 levels did not influence phosphorylation of Lhcb1 or Lhcb2 (Fig. 2A and B), nor the formation of the PSI-LHCI-LHCII complex in the *cB2.1* genotype (Fig. 3). Consistent with previous reports [22, 56, 57], we detected P-Lhcb2 in the PSI-LHCI-LHCII supercomplex, but not P-Lhcb1. These results emphasize the key role of Lhcb2 Thr-40 phosphorylation by STN7 in mediating ST [56, 57], since the mutation of this residue locked the plant in a state 1 condition, preventing the remodelling of the relative PSII/PSI antenna cross section and the dynamic re-equilibration of PSI/PSII excitation (Figs. 5 and 6). According to the structure of the PSI-LHCI-LHCII complex resolved via cryo-electron microscopy [69] and in line with spectroscopic investigations of ST [48], one trimer containing a single P-Lhcb2 polypeptide is sufficient to establish, and preserve, a stable direct connection of phosphorylated LHCII with the PSI PsaO subunit [45, 69]. In turn, PsaO mediates trimer association with the auxiliary PSI PsaL, PsaH and PsaI subunits [38, 59, 72, 110], enabling energy transfer from LHCII to the PSI core complex. In contrast, the mutation of the phosphorylatable Lhcb1 Thr-38 residue did not influence the above-mentioned mechanisms, as the *cB1.3*_*T38V*_ genotype was fully ST-competent, suggesting that P-Lhcb1 does not participate in the formation and/or stabilization of the PSI-LHCI-LHCII complex.

### The role(s) of Lhcb1 phosphorylation is a conundrum

**Fig. 8:**
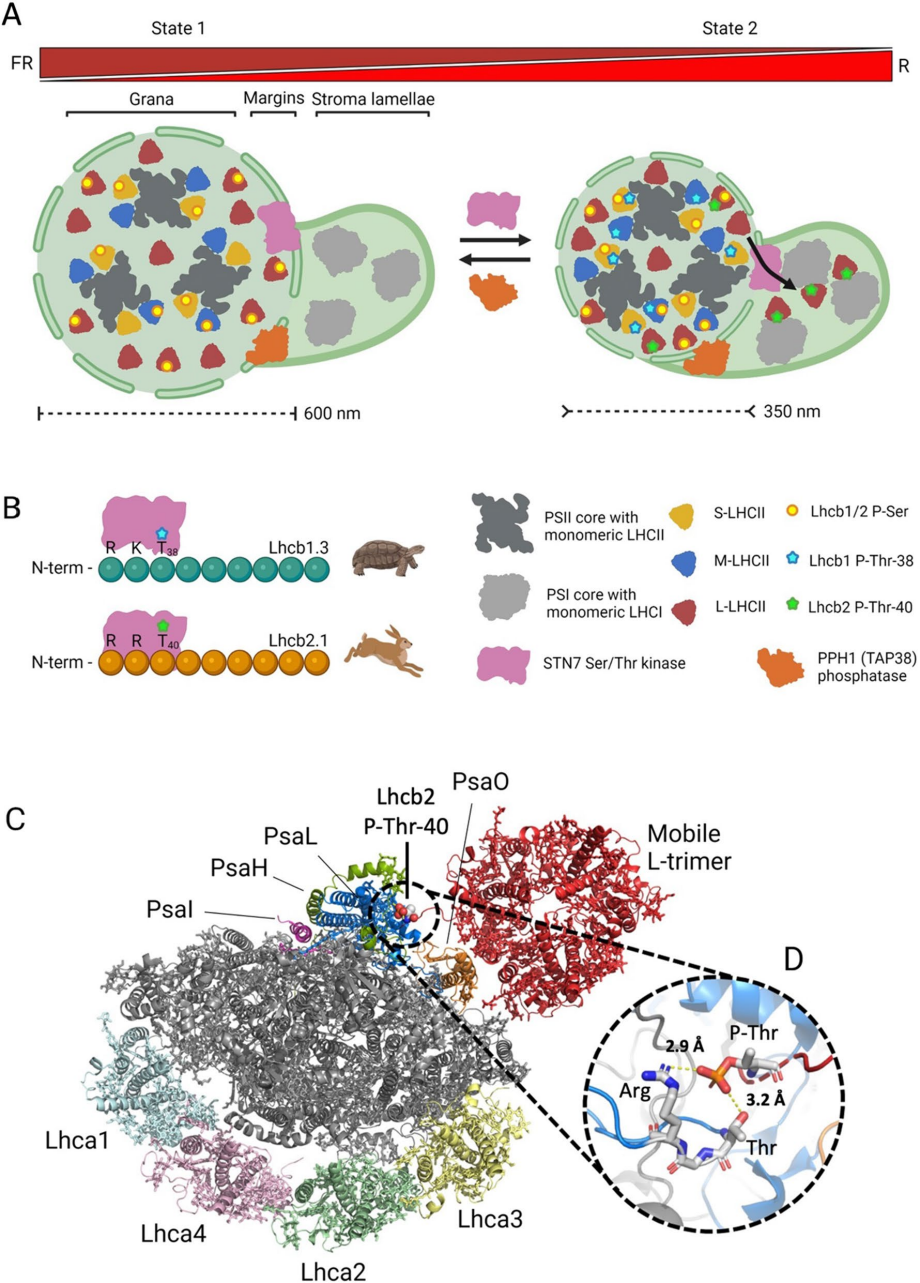
Comprehensive model explaining the roles of Lhcb phosphorylation during ST. Panel** A**: Top view of the thylakoid membrane system depicting the multiple consequences of Lhcb phosphorylation events. A shift of the light environment from a far red- (FR) enriched to a red- enriched (R) condition activates a state 1—state 2 transition involving the phosphorylation of threonine residues of the Lhcb1 and Lhcb2 polypeptides by the STN7 kinase. Under state 1, Lhcb1/2 serine residues are phosphorylated by a kinase unrelated to STN7 (yellow circles) and independently of LHCII trimer identity. Upon transition to state 2 light conditions, the activation of the STN7 kinase results in the phosphorylation of residues Thr-38 (cyan stars) and Thr-40 (green stars) of Lhcb1 (excluded the Lhcb1.4 isoform) and Lhcb2 polypeptides, respectively. The phosphorylated Lhcb1 polypeptides are exclusively found in the strongly- (gold) and moderately- (blue) bound LHCII trimers, while phosphorylated Lhcb2 polypeptides are enriched in the mobile L trimer type (red). The latter predominantly contains the non-phosphorylatable Lhcb1.4 protein isoform [32]. The phosphorylation of Lhcb proteins induces a shrinkage of the grana diameter which brings the L trimers in contact with the STN7 kinase resulting in further phosphorylation of Lhcb2 proteins. The phosphorylated L trimer pool migrates towards the stroma lamellae where it associates with PSI to form a PSI-LHCI-LHCII supercomplex. Upon shifts to state 1 light conditions, the PPH1 (TAP38) de-phosphorylates Lhcb1/2 proteins causing the return of L trimers to the grana region and association with PSII. The graphical elements displayed were created using the structures retrieved from the Protein Data Bank [16] of the following PBD files: 5XNL, stacked C_2_S_2_M2-type PSII-LHCII supercomplex from *Pisum sativum* [87], 5ZJI, photosystem I supercomplex with light-harvesting complexes I and II [69]. The structures of the Serine/threonine-protein kinase STN7(Uniprot sequence code Q9S713) and TAP38 protein phosphatase (Uniprot sequence code P49599) were created with the AlphaFold Protein Structure software [94]. Panel** B**: the faster phosphorylation kinetics of Lhcb2 polypeptides compared with Lhcb1 by STN7 are explained by an optimal recognition of the substrate by the kinase owing to two consecutive arginine (R) residues directly upstream of the phosphorylatable Thr-40 residue [54]. The figures displayed in panel A and B were created with BioRender.com. Panel** C**: tridimensional structure of the PSI-LHCI-LHCII supercomplex of *Zea may*s*,* (PDB file 5ZJI) [69] depicting the PSI antenna system composed of the Lhca1 (cyan), Lhca4 (pink), Lhca2 (light green), Lhca3 (yellow) polypeptides in association with the phosphorylated LHCII L trimer (red). The PSI subunits which mediate the interaction are highlighted: PsbO (orange); PsbL (blue), PsbH (green), and PsbI (magenta). Panel** D**: detailed region occupied by the phosphate group of Lhcb2 P-Thr-40 and of its stabilizing interactions implicated in the formation of the PSI-LHCI-LHCII supercomplex mediated via hydrogen bonds (dotted yellow lines) with the amino group of arginine and the hydroxy group of threonine residues of the PSI PsbL subunit

This work confirmed that Lhcb1 (1.3) residue Thr-38 is a light-dependent substrate of the STN7 kinase (Fig. 2) [52, 71] and, yet, this phosphosite is not essential for the formation of the PSI-LHCI-LHCII supercomplex (Fig. 3), nor to promote PSII fluorescence quenching upon state 1—state 2 transition (Figs. 5 and 6). Our data, thus, question the relevance of Lhcb1 phosphorylation in the process of ST and call for revisiting its functional implication(s) in a broader physiological context. It should considered that the mobile (L) LHCII trimers that transiently associate with PSI are enriched in the Lhcb1.5 and Lhcb1.4 isoforms [32, 50], the latter lacking the phosphorylatable Thr residue at position 38. Indeed, in agreement with previous work (Paolo [56, 57], we did not detect P-Thr-38-Lhcb1 epitopes in the PSI-LHCI-LHCII band (Fig. 4), but only in the higher molecular weight PSII supercomplexes and megacomplexes. These latter mostly consist of moderately-(M) and strongly-(S) bound trimers that are not displaced during ST due to their higher binding affinity to the PSII core complex [13, 98, 99]. Of note, while M trimers are strongly enriched in the Lhcb3 isoform and do not contain Lhcb2 proteins, the S ones contain fairly equal amounts of Lhcb1.1–3 and Lhcb2 isoforms [32, 50]. The diverse configurations in which LHCII trimers are assembled is a clear example of sub-functionalization of Lhcb proteins, each being a unique biological entity endowed with specialized roles [6, 23]. At the same time, the uneven and seemingly random distribution of trimers in different thylakoid regions [90] likely holds a functional relevance, to promote the dynamic association/dissociation of the mobile LHCII pool between PSII and PSI. Accordingly, it was reported that under state 2 conditions, the PSI-associated LHCII pool exhibits a higher phosphorylation level (≈ 40%) compared with the non-dissociated PSII-bound pool (≈ 15%) [22]. Also, the appressed grana stacks are less phosphorylated than the grana margins due to the selective loss of the P-Lhcb2 pool upon migration of L trimers to the stroma lamellae. While the P-Lhcb2 pool selectively populates the grana margins and stroma lamellae, the phosphorylation profile of Lhcb1 exhibits a homogeneous distribution in the thylakoid system [22]. These conclusions are consistent with the mobile LHCII pool being selectively enriched in the non-phosphorylatable Lhcb1.4 isoform and because of the equal contribution of phosphorylatable Lhcb1 isoforms to the non-mobile M and S trimers [32, 50]. The differential localization of P-Lhcb2 and P-Lhcb1 in state 2 (Fig. 4) is fully compatible with the lateral heterogeneity of thylakoid membranes, with PSII enriched in the grana stacks, while PSI confined to the stroma lamellae and grana margins [4], together with the STN7 kinase (Tobias [105, 106]. This spatial segregation implies that the mobile L trimer sub-population(s) might reside in proximity of the STN7 kinase (Fig. 8). Taken together, these observations are consistent with the faster phosphorylation kinetics of Lhcb2 vs. Lhcb1 reported by [52]. Also, the identity of two positively charged residues upstream of the phosphosite appears to affect the interaction with STN7 and phosphorylation kinetics as reported from in vitro mutational analysis [54]. Indeed, Thr-38 in Lhcb1 is preceded by arginine (R) and lysine (K) residues, while Lhcb2 Thr-40 carries two consecutive Rs which appear to promote optimal substrate recognition and faster phosphorylation kinetics by STN7 (Fig. 8 panel B). It is noteworthy that both Lhcb1 Thr-38 and Lhcb2 Thr-40 residues from *Arabidopsis thaliana* exhibit a strong phylogenetic conservation across land plants (Additional file 1: Figs. S8 and S9). However, some species which are adapted to light environments that are either constitutively enriched or depleted in far-red radiation, such as *Pteris vittata, Sphagnum fallax or Zostera marina,* lack the corresponding Thr sites of Lhcb1 and Lhcb2, or both, and were reported to display low fluorescence quenching amplitude upon state 1—state 2 transition [20]. For instance, the shade-tolerant fern *Pteris vittata* lacks both residues, while the Thr of Lhcb1 is missing in the moss *Sphagnum fallax* (position occupied by a phosphorylatable serine) and in the angiosperm eelgrass *Zostera marina*.

### The dark side of LHCII serine phosphorylation

Although this work focussed on the role(s) of consensus Lhcb Thr residues in the context of ST, we highlighted the occurrence of LHCII serine phosphorylation events that are STN7- and light-independent (Fig. 7B). To our knowledge, this topic has received considerably less attention compared with the light-dependent phosphorylation of chloroplast proteins. Notably, two P-Ser have been experimentally assigned with high-confidence to the same Lhcb1 tryptic peptide (GPSG(pS)PWYG(pS)DRVK): P-Ser 48 (46 in Lhcb1.5) [19, 64, 78, 88, 96, 97, 107] and P-Ser 53 (51 in Lhcb1.5). Together with P-Thr-38, P-Ser 48 and P-Ser 53 form a phosphorylation hotspot at the N-terminal end of all Lhcb1 isoforms (except for Lhcb1.4) [34], indicating that this region is the substrate of multiple, differentially regulated kinases [82],Tobias [105, 106]. Both P-Ser sites are detected with higher frequency than P-Thr 38 (> 20 times higher number of observations) according to the *Arabidopsis* PeptideAtlas database (www.peptideatlas.org/builds/arabidopsis/) [93, 100], suggesting the existence of a constitutive, light-independent LHCII phosphorylation state of elusive physiological significance. Similarly, the experimentally described Lhcb2 P-Ser 43 contained in the tryptic peptide (pS)TPQSIWYGPDRPK [78] is observed more frequently than Lhcb2 Thr-40, but with lower frequency (< 10 times less) than Lhcb1 P-Ser 48 and P-Ser 53. Although STN7 is described as a serine-threonine kinase [92, 101], and responds to the reduction of plastoquinone in the dark [41], we exclude this kinase as effector of Ser phosphorylation since LHCII serine phosphorylation was observed in the *kostn7* mutant (Fig. 7B). The STN8 kinase, instead, could be a potential candidate for these light-independent phosphorylation events [25, 58, 82, 105, 106]. Moreover, STN8 is known to phosphorylate the PSII core protein CP43 independently of light [29], beside its high light-dependent activity [14], suggesting an extended metabolic regulation of the thylakoid phosphorylation networks. Three out of the 15 protein kinases localized to the chloroplast [10] are integral membrane proteins: STN7, STN8 and the PLASTID CASEIN KINASE II (pCKII) [55]. pCKII is known to target components of the plastid transcriptional machinery [53] independently of light, thus, pCKII is a candidate kinase for the phosphorylation of LHCII serine(s) in the dark.

### An integrated model for Lhcb phosphorylation in state transitions

The experimental evidence provided in this work warns against a simplistic interpretation of Lhcb1 and Lhcb2 phosphorylation as merely synergistic events in the process of ST, giving way to speculative scenarios explaining key events leading to re-equilibration of the relative PSI/PSII excitation pressure. It is estimated that the L trimer population accounts for ≈ 50% of the entire LHCII pool [1]. Of this fraction ≈ 35% undergoes reversible association/dissociation between PSII and PSI upon light quality shift [67]. In most light conditions, however, a LHCII fraction stably associates with PSI serving as shared antenna system [99] in a so-called “energetically connected lake” [35]. This configuration relies on a basal LHCII phosphorylation level which, according to the light condition, is dynamically modified by the antagonistic activities of the STN7/TAP38 pair [36]. The structural consequences of Lhcb phosphorylation have been thoroughly investigated and are key to understand the transient formation of PSI-LHCII associations during ST [3]. According to a previously proposed working model, structural reshaping of the PSII supercomplex precedes—and facilitates—the occurrence of ST [28]: during state 1, several PSII core components are phosphorylated by multiple kinases, possibly including STN8 kinase, enabling the formation of megacomplexes including several PSII dimers [75]. This stable configuration requires the PsbW (García-Cerdán et al. 2011) and Psb27 [28] PSII subunits, as their respective mutants exhibited faster ST kinetics. This effect is likely explained by a weaker association of S and M trimers to the PSII core complex, suggesting that a “molecular brake”, possibly relying on phosphorylation of serine residues of Lhcb proteins (Fig. 7B), is at play to prevent LHCII-PSI association and, thus, wasteful energy transfer (spillover) between the two photosystems. Upon shift to state 2 light and concomitant activation of STN7, the PSII core CP43 protein gets phosphorylated, leading to rapid supercomplex disassembly. The disassembly of PSII supercomplexes is believed to promote trimer mobility towards the grana margins where they encounter the STN7 kinase and get phosphorylated [29, 31], which entrains a reduction of the number of grana stacks per chloroplast and the shrinking of grana [102] causing a reduction of grana diameter upon state 1—state 2 transition (≈ 580 nm in state 1 vs. 360 nm in state 2) [102] (Fig. 8, panel A). This event appears to favour the diffusion of plastoquinol and, thus, PSI reduction [40], but also brings L trimers closer to the grana margins where they are further phosphorylated by STN7 and more easily migrate towards the stroma lamellae where they associate with PSI [103]. The molecular details underlying the docking of the phosphorylated L trimer to PSI have been resolved through cryo-electron microscopy revealing the positioning of the phosphate moiety attached to Lhcb2 Thr-40 at the interface between the L trimer and the PSI PsaL subunit [69] (Fig. 8, panel C). The phosphate moiety interacts with the amine group of arginine 174 and the hydroxyl group of threonine 172 of the PsaL polypeptide establishing two hydrogen bonds (yellow dotted lines in panel D of Fig. 8) that stabilize the association between the LHCII trimer and PSI, enabling the formation and maintenance of the PSI-LHCI-LHCII supercomplex.

### A qT component independent from Lhcb2 Thr-40 phosphorylation

Although the phosphorylation of Lhcb2 Thr-40 is required for the formation of PSI-LHCI-LHCII supercomplex and accounts for ≈ 60% of qT (Figs. 5 and 6), a residual qT was observed in both the *cB2.1*_*T40V*_ and *koLhcb2* genotypes. This fluorescence decay appears to be a genuine component of state 1—state 2 transition since it is light- and STN7-dependent, and is required for the full re-equilibration of chloroplast redox state (Fig. 5A and B). Despite it is tempting to attribute this 40% residual quenching to Lhcb1 phosphorylation, this hypothesis is contradicted by the full qT activity (Fig. 5A) and excellent redox re-equilibration of both *cB1.3* and *cB1.3*_*T38V*_ genotypes. Figure 7A and B, moreover, show that no other light-dependent and STN7-dependent phosphorylation event occurs at either Ser or Thr sites. Beside LHCII, the PSII core subunits D1 and D2 are also phosphorylated; yet maximal D1 + D2 phospho-signal is enhanced in the only condition in which the residual qT component was inactive, *i.e.* in the *kostn7* mutant (Fig. 5A, B, *kostn7* lane). One possibility is that this **r**esidual qTr relies on the synergistic phosphorylation of both Lhcb1 and Lhcb2. This is consistent with the observation that the *cB2.1*_*T40V*_ and *koLhcb2* genotypes exhibit partial re-equilibration of their redox state. How this could be realized at the molecular level? The disconnection of mobile L trimers from PSII supercomplex in the grana could make available a docking site for PSI-LHCI for energy spillover, either directly, or by intermediation of LHCII trimers not involved in the formation of digitonin-resistant PSI-LHCI-LHCII complexes (Fig. 3). Such LHCII trimers have been reported based on spectroscopic and genetic evidence, and can be either phosphorylated or not [12, 81], while the formation of PSI-PSII megacomplexes has been reported by several authors [49, 76] and suggested to be located in the grana margins [108, 109]. Fast-spectroscopy studies during state1-state 2 transitions are thus needed to elucidate whether the qTr in the *koLhcb2* and *cB2.1*_*T40V*_ genotypes relies on PSI or on a decrease in fluorescence lifetime of LHCII itself. Finally, this component might be caused by the light-dependent phosophorylation of non-Lhcb substrate(s) by STN7. Potential candidates include the CURVATURE THYLAKOID 1 (CURT1) proteins, which are known to be part of the STN7/STN8 chloroplast kinase network [91] and influence thylakoid architecture dynamics [5, 74]. Intriguingly, the depletion of CURT impairs photosynthetic acclimation *Arabidopsis* in response to light quality shifts by limiting the amplitude of state 1—state 2 transitions [74].

## Conclusions

Despite over 40 years of studies of ST since their first description in a green microalga [15], the molecular details underlying this photosynthetic acclimation mechanism in plants have not yet reached a full understanding. Recent seminal studies based on RNA antisense-based suppression of Lhcb protein synthesis [71] have proposed complementary roles for Lhcb1 and Lhcb2 polypeptides during ST. Moreover, according to their different phosphorylation kinetics [52], it was concluded that Lhcb2 is the key player in the dynamic equilibration of the relative PSI/PSII excitation pressure upon shifts in light quality, while the more abundant Lhcb1 pool is crucial in the phosphorylation-dependent regulation of thylakoid ultrastructure. Although we could fully confirm the essential role of Lhcb2 Thr-40 phosphorylation during ST, we found no evidence for a contribution of Lhcb1 Thr-38 to this process, since neither the amplitude, nor the kinetics of fluorescence quenching were affected by its substitution in vivo with a non-phosphorylatable residue. We also showed that a relevant component of qT (≈ 40%) is independent from Lhcb2 phosphorylation, and requires future analysis to identify its molecular nature. A future step to verify a potential synergistic contribution of Lhcb1 and Lhcb2 phosphorylation to the residual qT could the co-complementation of their non-phosphorylatable forms. In conclusion, this work provided novel insights into the consequences of individual Lhcb phosphorylation events during ST and opens the way to engineer this mechanism in crop biotechnology, with respect to dense cultivation settings where shading inside canopies is major limiting factor yield potential.

## Materials and methods

### Plant material, growth conditions and production of *Lhcb1* and *Lhcb2* knockout genotypes

*Arabidopsis thaliana* wild type (Col-0) and the previously described *stn7* mutant line (SALK_073254) [11] were used as positive and negative controls in all experiments. The *koLhcb1* and *koLhcb2* described in this work lines were developed using a multiplex CRISPR-Cas9-based genome editing tool using previously described gRNAs arrays designed with the CRISPOR online tool [21] to target all five *Lhcb1* [66] and three *Lhcb2* gene isoforms [37] clades. Complementation of *koLhcb1* and *koLhcb2* backgrounds with wild type and phosphomutant *lhcb1.3* and *lhcb2.1* versions was carried out via the floral dip transformation protocol using *Agrobacterium tumefaciens* strain GV3101 [111] followed by selection of T1 transformant seedlings on MS agar medium supplemented antibiotics (kanamycin 50 mg ml^−1^ and hygromycin 25 mg ml^−1^ for *lhcb1.3* and *lhcb2.1* complementation, respectively). All genotypes were grown in soil under controlled conditions using a white neon light source of 100 μmol photons m^−2^ s^−1^ at 23 °C, 70% relative humidity under short day (8 h light/16 h dark) photoperiod. The light spectrum was measured with a SpectraPen Mini device (Photon System Instruments, Drásov, Czech Republic (shown in Additional file 1: Fig. S1). All physiological biochemical and biophysical analyses were performed on 6 weeks old plants (Additional file 1: Tables S1 and S2).

### Assembly of complementation vectors

To reintroduce selected *Lhcb1* and *Lhcb2* gene isoforms in the respective *koLhcb1* and *koLhcb2* lines, complementation vectors were created using standard restriction enzyme and ligation- based molecular cloning techniques. The *Lhcb1.3* (AT1G29930) gene was chosen as representative member of the *Lhcb1* gene family to re-establish wild type Lhcb1 protein levels being the most highly expressed among *Lhcb1* isoforms [43]. Following the same rationale, the *Lhcb2.1* (AT2G05100) gene was selected as representative *Lhcb2* isoform. In the case of the *Lhcb1.3* gene, a portion corresponding to 958 bp upstream to the start codon extending into the coding sequence and the 3′ UTR (total length of 2002 bp) was amplified via Polymerase Chain Reaction (PCR) with a DNA polymerase with proofreading activity (Hybrid DNA polymerase, EURx, Gdánsk, Poland) using the oligonucleotide pair FW-XmnI-P-Lhcb1.3 CAGGTCTAAGAAAATATTCCTGAAG and RV-KpnI-3′UTR-Lhcb1.3 GGGGTACCACAAATGTGTTTGATTTGTACGGAT. The purified PCR product ligated into the pH7WG2 GATEWAY™ vector backbone [47] previously digested with the restriction enzyme pair PmeI and KpnI (New England Biolabs, Ipswich, MA, USA) to create the complementation vector *pH7WG2-Lhcb1.3*. For the *Lhcb2.1* gene, the spliced, intronless coding sequence and its native 5′- and 3′ UTRs were amplified from *A. thaliana* cDNA produced from total RNA previously extracted with the NucleoZol reagent kit (Macherey–Nagel AG, Germany). The PCR product (total length 1150 bp) was obtained with oligonucleotides FW-KpnI-3′UTR-Lhcb2.1 CACTTACTTACACCCTCGTGAC and RV-SpeI-5′UTR-Lhcb2.1 GGACTAGTGTTGTTGTAAGCCAA. Next, the *Lhcb1.3* promoter was amplified via PCR from the *pH7WG2-Lhcb1.3* plasmid using the oligonucleotide pair FW-XbaI*-P-Lhcb1.3* GCTCTAGATGAACGCCTTCTCTG and RV-KpnI-*P-Lhcb1.3* GGGGTACCCGTGTCCAGGCCTACTTTTACG, and subsequently ligated in vitro together with the *Lhcb2.1* PCR-amplified fragment using a T4 ligase enzyme (New England Biolabs)*.* The ligation product was cloned in the pK7WG2 GATEWAY™ vector backbone [47], previously digested with the restriction enzyme pair PmeI and KpnI (New England Biolabs, Ipswich, MA, USA) to create the complementation vector *pK7WG2-Lhcb2.1*.

Site-directed mutagenesis was conducted on the *pH7WG2-Lhcb1.3* and *pK7WG2-Lhcb2.1* vectors using oligonucleotides mismatch-containing to enable the modification of triplets coding for ammino acid residues corresponding to phosphorylatable residues using the Q5 site-directed mutagenesis kit (New England Biolabs, Ipswich, MA, USA). Threonine 38 and threonine 40 of the Lhcb1.3 and Lhcb2.1 polypeptides, respectively, were selected as candidate residues based on experimental evidence pointing to their phosphorylated status in vivo [34]. Furthermore, these residues are recognized as part of phosphorylated epitopes of the Lhcb1.3 and Lhcb2.1 polypeptides (RKT*VAKPKGP and RRT*VKSTPQS, respectively, asterisk refers to phosphate group position) by α-P-Lhcb antibodies [52] used in this work. Mutagenetic primers were designed using the NEBaseChanger online tool (https://nebasechanger.neb.com/). The following oligonucleotide pairs *Lhcb1.3T38V-FW* AATGAGGAAGgtTGTTGCCAAGCCAAAGGGC/*Lhcb1.3T38V-RV* GTCACACGGCCGCTTCCA and *Lhcb2.1T40V-FW* CATGCGTCGTgttGTCAAGTCTACTCCTCAAAGCATC/*Lhcb2.1T40V-RV* GTCACACGGCCACCACCG were used to introduce the desired mutations in the *Lhcb1.3* and *Lhcb2.1* sequences, respectively, to create the *pH7WG2-Lhcb1.3*_*T38V*_ and *pK7WG2-Lhcb2.1 *_*T40V*_ vectors.

### Denaturing SDS-PAGE and immunoblotting

All SDS-PAGE experiments were performed using the Tris-Tricine buffer gel system [80]. In the case of immunodecoration experiments with total leaf protein extracts, 0.5 μg of Chl (unless otherwise stated) were separated on SDS-PAGE and subsequently electroblotted on nitrocellulose membranes. Quantitative western blots, instead, were performed on isolated thylakoids [44] using Chl ranges spanning between 0.10 and 0.45 μg. All membranes were incubated with primary antibodies against α-PsbB/CP47 (AS04 038, Agrisera, Vännäs, Sweden), α-Lhcb1 (AS01 004), α-Lhcb2 (AS01 003), α-STN7 (AS16 4098) and either anti rabbit alkaline phosphatase (AP)-conjugated (Sigma-Aldrich A3687) or horse radish peroxidase (HRP)-conjugated (AS10 668), and anti-mouse AP-conjugated (Sigma-Aldrich A9044) secondary antibodies. The previously described α-P-Lhcb1 (AS13 2704), α-P-Lhcb2 (AS13 2705) were used to specifically probe the phosphorylation status of Lhcb1/2 proteins following a 2-h treatment with PSII-favoring (state 2-inducing) light. The global phosphorylation status of thylakoid proteins was assessed using α-phospho-threonine (Cell Signaling Technology, Danvers, USA, #9386) and α-phospho-serine (AS20 4487) antibodies. Blots were developed with a standard colorimetric protocol or via enhanced chemiluminescence method. Signal amplitude/strength was quantified using the GelPro 3.2 software (Bio-Rad, Hercules, CA, USA).

### Separation and biochemical characterization of native thylakoid protein complexes

Non-denaturing large pore blue native gels (lpBN) were prepared according to an established protocol [44]. Briefly, intact, stacked thylakoids were prepared from plants following 2-h adaptation to PSII-favoring (state 2-inducing) light (≈ 50 μmol photons m^−2^ s^−1^) or overnight dark incubation (state 1). All buffer systems included the protease inhibitors phenylmethylsulfonyl fluoride (PMSF, 100 μM) and aminocaproic acid (500 μM), and the phosphatase inhibitor NaF (10 mM) to preserve the phosphorylated status of thylakoid proteins. Solubilization of thylakoids was performed starting from 40 μg of Chl by shortly (10 s) vertexing the samples in the presence of 1% (w/v) digitonin, followed by 20 min incubation at 4 °C under gentle agitation. For immunodecoration of native protein complexes, lpBN gels were first incubated for 2 h in a denaturating buffer (20 mM Tris, 125 mM Glycine, 6 M Urea, 2% SDS) then washed 3 times in DD water and blotted for 2 h following the same procedure described above. Non-denaturing Deriphat-PAGE experiments gels were performed with stacked thylakoids prepared from 35 ug Chl according to [18] and following an established protocol [70] using the following gel system: stacking 3.5% (w/v) acrylamide 48/1.5 (48% acrylamide/1.5% bisacrylamide), 12 mM Tris, 48 mM glycine, pH 8.5; resolving: acrylamide (48%/1.5%) gradient from 4 to 8% (w/v) stabilized by a glycerol gradient from 8 to 16%, 12 mM Tris, 48 mM glycine, pH 8.5. Gel polymerization was conducted with 0.02% ammonium persulfate and 0.075% TEMED. Gels were run overnight at 4 °C under constant voltage of 50 V using an electrophoresis reservoir buffer composed of: 12.4 mM Tris, 96 mM glycine, pH 8.3, and purified 0.1% Deriphat-160. Electrophoresed overnight [39].

### Spectroscopic analysis of pigment composition

Pigments were extracted from dark adapted leaves discs using 80% acetone buffered with Na_2_CO_3_.

Absorption spectra were recorded at RT using a Aminco DW-2000 spectrophotometer. Leaf pigment content, Chl a/b ratio and Chl/Car ratio were calculated from the spectra obtained from acetonic extracts of 5 biological replicates following an established method [24].

### Room temperature PSII chlorophyll fluorescence analysis

The standard Photosynthetic parameters Fv/Fm, qP and qL [7] were derived from the room temperature analysis of leaves using a Dual PAM-100 fluorimeter (Walz,). State 1—state 2 transition kinetics were assessed in three biological replicates for each genotype using an established protocol [59] consisting of a first interval of 15 min of low intensity actinic light (AL, 50 μmol photons m^−2^ s^−1^) followed by the superimposition of far-red (FR) light to induce state 1 for 15 min and a final AL interval of similar duration. The qT (ST-dependent quenching) parameter, which reflects the amplitude of PSII cross section change, was calculated as (Fm′ − Fm″) Fm′ · 100, where Fm′/′′ are the maximal fluorescence yield values at the end of state 1–2 intervals, respectively [26]. The 1-qP and the analogous 1-qL parameter [51] were obtained in correspondence of the saturating pulses employed to determine the maximum PSII fluorescence emission (Fm’ and Fm’’) measured at the end of state-1 and state-2 intervals, respectively. The functional PSII antenna size was measured in a home-built Chl fluorimeter using a dim green light (10 μmol photons m^−2^ s^−1^) [77] in dark-adapted leaves infiltrated with 3-(3,4-dichlorophenyl)-1,1-dimethylurea, (DCMU, 50 μM).

### Supplementary Information

**Additional file 1**.** Fig. S1** spectrum of growth and state 2-inducing light.** Fig. S2** functional PSII chlorophyll antenna size of wild type and koLhcb1, koLhcb2 genotypes. The functional PSII antenna size measured using a dim green light (10 μmol photons m^−2^ s^−1^) in dark-adapted leaves infiltrated with DCMU (50 μM). The functional antenna size is estimated as the reciprocal of T2/3 of the Chl fluorescence rise.** Fig. S3** Coomassie-stained SDS-PAGE of thylakoids from background genotypes and complemented lines.** Fig. S4** Deriphat-PAGE of knockout and complemented genotypes created in this work.** Fig. S5** Immunological characterization of knockout background genotypes and of complemented lines.** Fig. S6** densitometric quantification of STN7 protein levels in the complemented lines.** Fig. S7** Fluorescence traces of all genotypes recorded during the PAM state transitions protocol.** Table S1** list of amino acid sequences of Lhcb1.3 orthologs of species from different taxonomic/phylogenetic groups used for multiple sequence analysis.** Table S2** list of amino acid sequences of Lhcb2.1 orthologs of species from different taxonomic/phylogenetic groups used for multiple sequence analysis.

## Data Availability

Genotypes and sequence data employed in this work are available in the *Arabidopsis* Genome Initiative or GenBank/EMBL databases under accession numbers At1g68830 (Stn7), At1g29920 (Lhcb1.1), At1g29910 (Lhcb1.2), At1g29930 (Lhcb1.3), At2g34430 (Lhcb1.4), At2g34420 (Lhcb1.5), At2g05100 (Lhcb2.1), At2g05070 (Lhcb2.2), At3g27690 (Lhcb2.3) and At5g54270.
