# Supplementary material for: Analysis of state 1—state 2 transitions by genome editing and complementation reveals a quenching component independent from the formation of PSI-LHCI-LHCII supercomplex in Arabidopsis thaliana
Source: Biol Direct. 2023 Aug 23;18:49. doi: 10.1186/s13062-023-00406-5 (PMC10463614; doi:10.1186/s13062-023-00406-5)
Supplement: Supplementary file 1 — Additional file 1. Fig. S1 spectrum of growth and state 2-inducing light. Fig. S2 functional PSII chlorophyll antenna size of wild type and koLhcb1, koLhcb2 genotypes. The functional PSII antenna size measured using a dim green light (10 μmol photons m−2 s−1) in dark-adapted leaves infiltrated with DCMU (50 μM). The functional antenna size is estimated as the reciprocal of T2/3 of the Chl fluorescence rise. Fig. S3 Coomassie-stained SDS-PAGE of thylakoids from background genotypes and complemented lines. Fig. S4 Deriphat-PAGE of knockout and complemented genotypes created in this work. Fig. S5 Immunological characterization of knockout background genotypes and of complemented lines. Fig. S6 densitometric quantification of STN7 protein levels in the complemented lines. Fig. S7 Fluorescence traces of all genotypes recorded during the PAM state transitions protocol. Table S1 list of amino acid sequences of Lhcb1.3 orthologs of species from different taxonomic/phylogenetic groups used for multiple sequence analysis. Table S2 list of amino acid sequences of Lhcb2.1 orthologs of species from different taxonomic/phylogenetic groups used for multiple sequence analysis. [file 13062_2023_406_MOESM1_ESM.docx]

**Supplementary information**

**
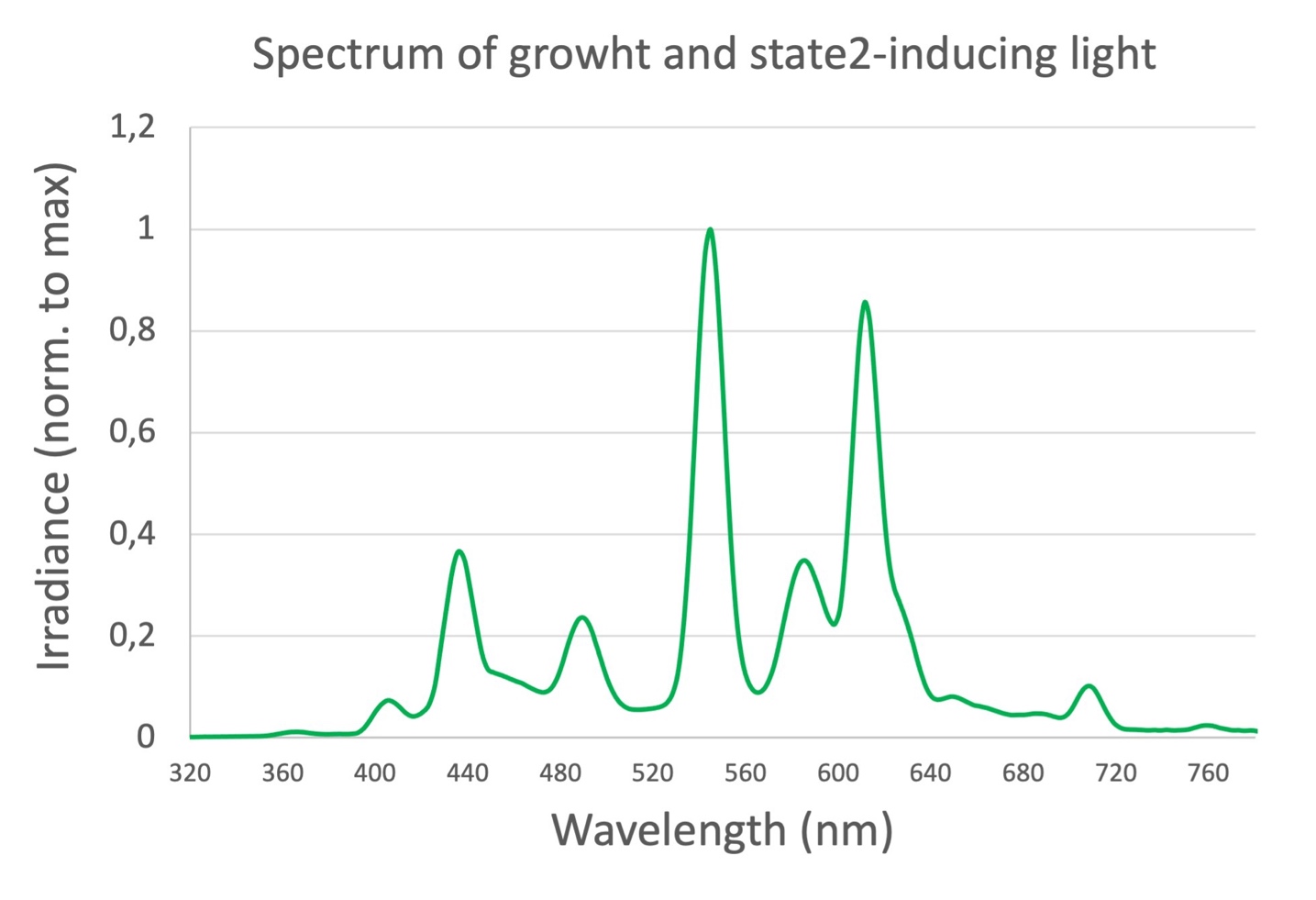
**

**Figure S.1: spectrum of growth and state 2-inducing light.**

**
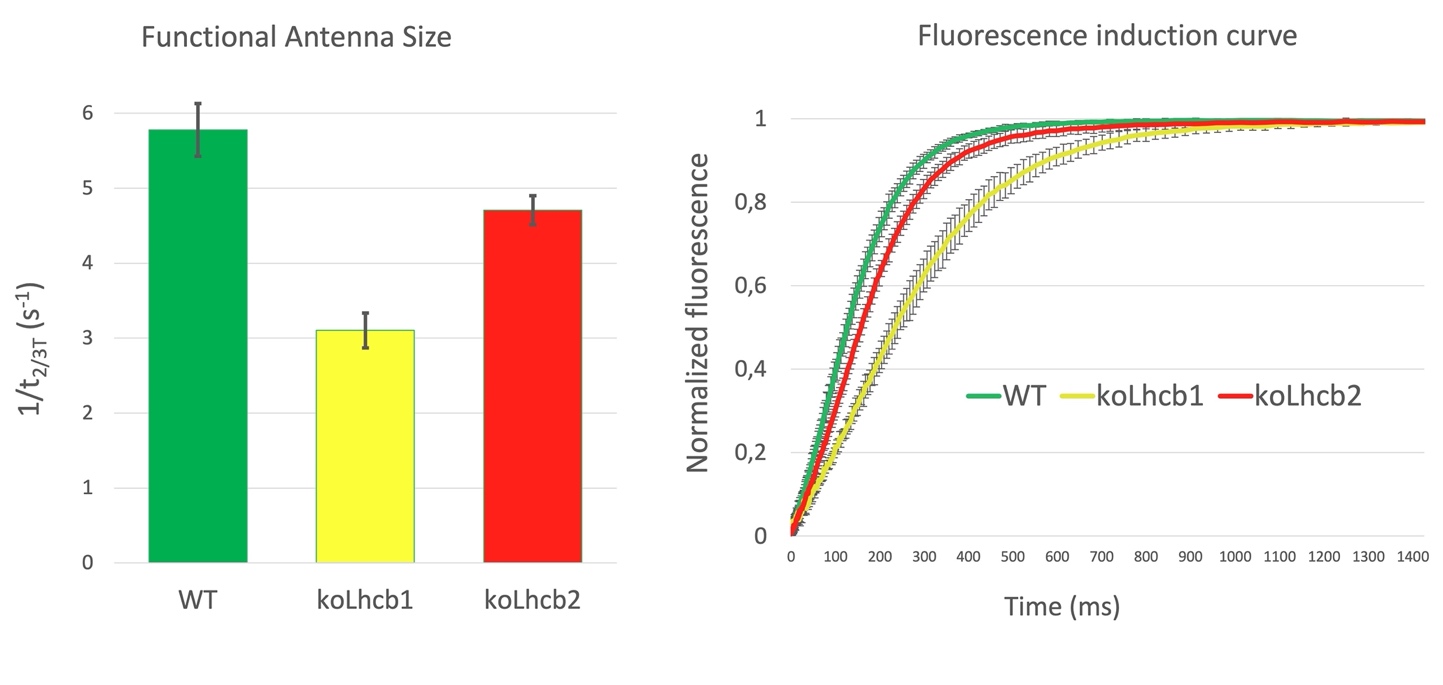
**

**Figure S.2: functional PSII chlorophyll antenna size of wild type and *koLhcb1*, *koLhcb2* genotypes.**

The functional PSII antenna size measured using a dim green light (10 μmol photons m^−2^ s^−1^) in dark-adapted leaves infiltrated with DCMU (50 μM). The functional antenna size is estimated as the reciprocal of T_2/3_ of the Chl fluorescence rise.

**
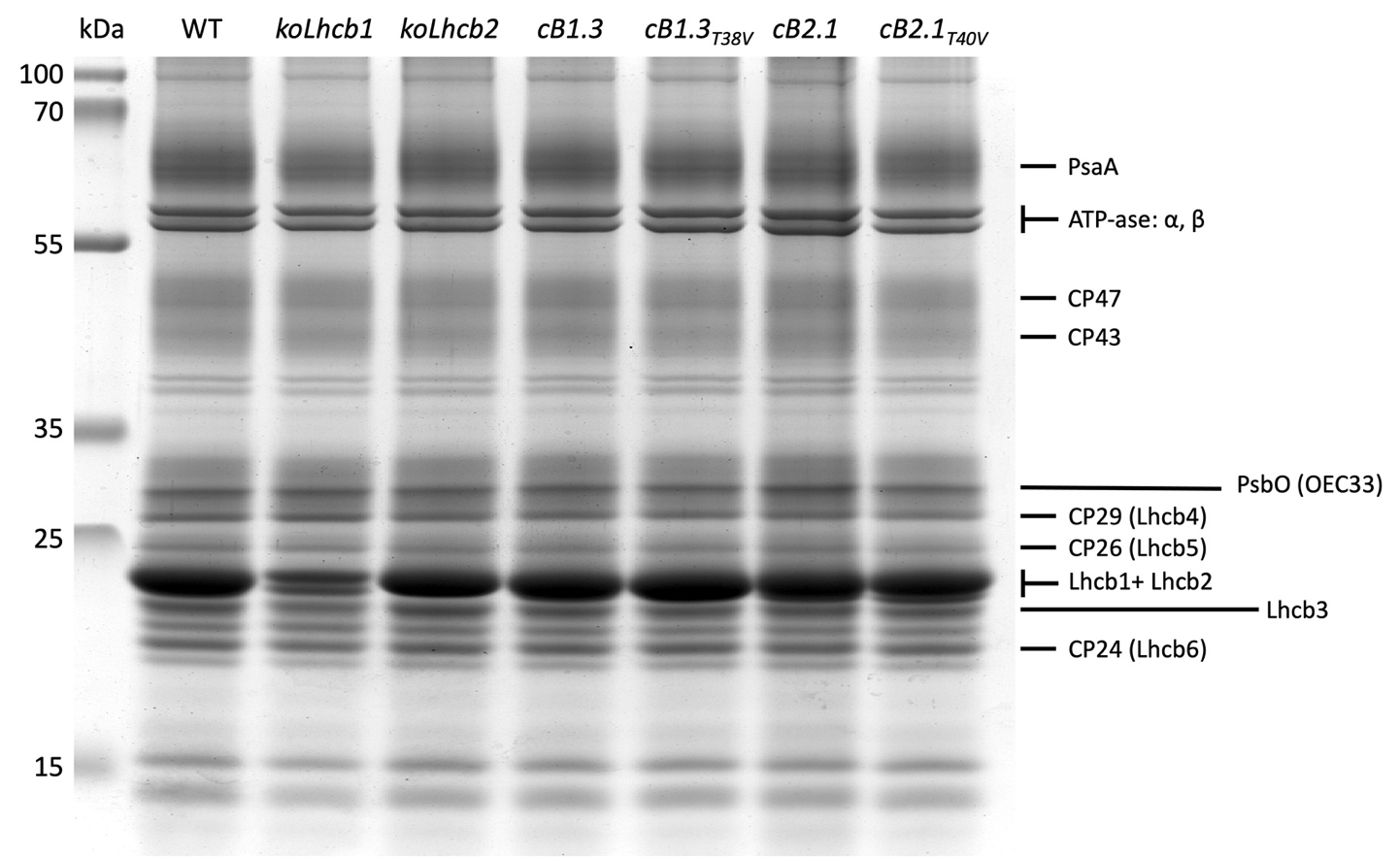
**

**Figure S.3: Coomassie-stained SDS-PAGE of thylakoids from background genotypes and complemented lines.**

**
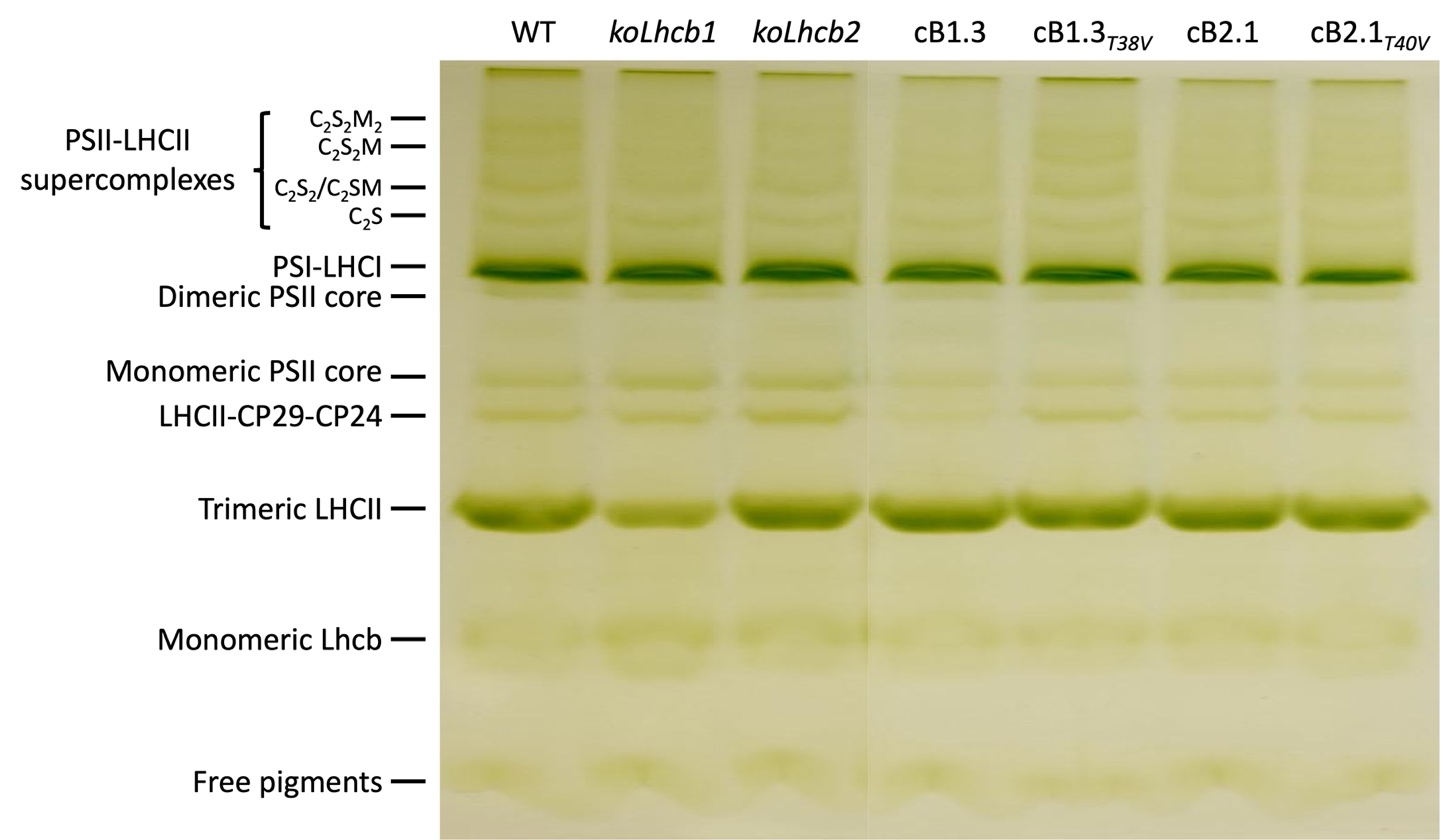
**

**Figure S.4: Deriphat-PAGE of knockout and complemented genotypes created in this work.**

**
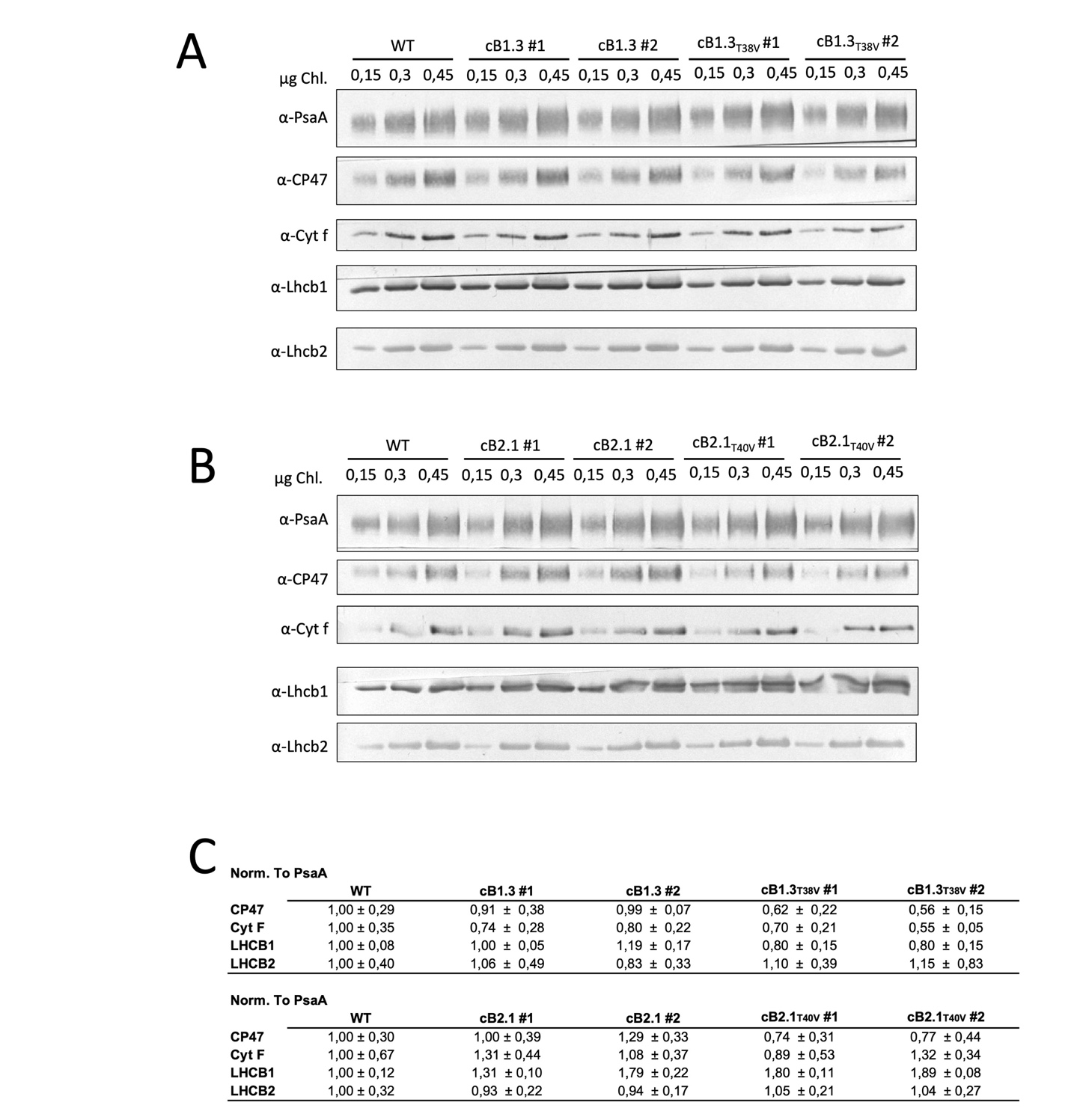
**

**Figure S.5: Immunological characterization of knockout background genotypes and of complemented lines.**

**
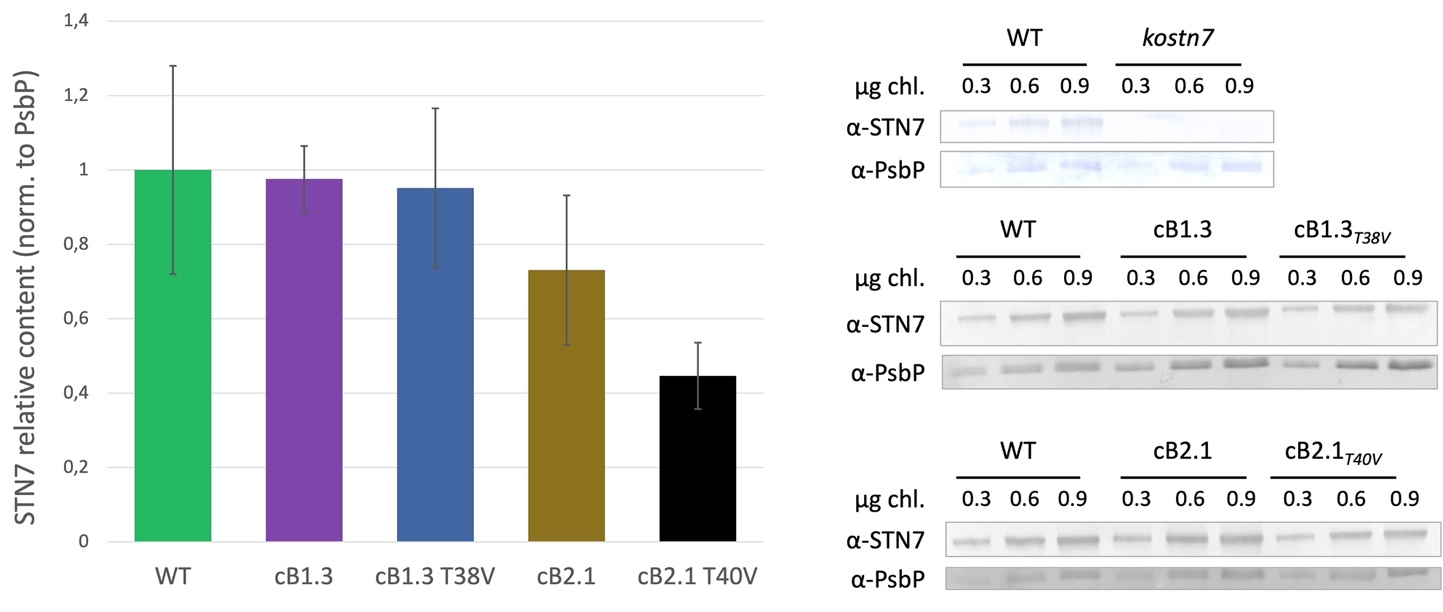
**

**Figure S.6: densitometric quantification of STN7 protein levels in the complemented lines**.

**
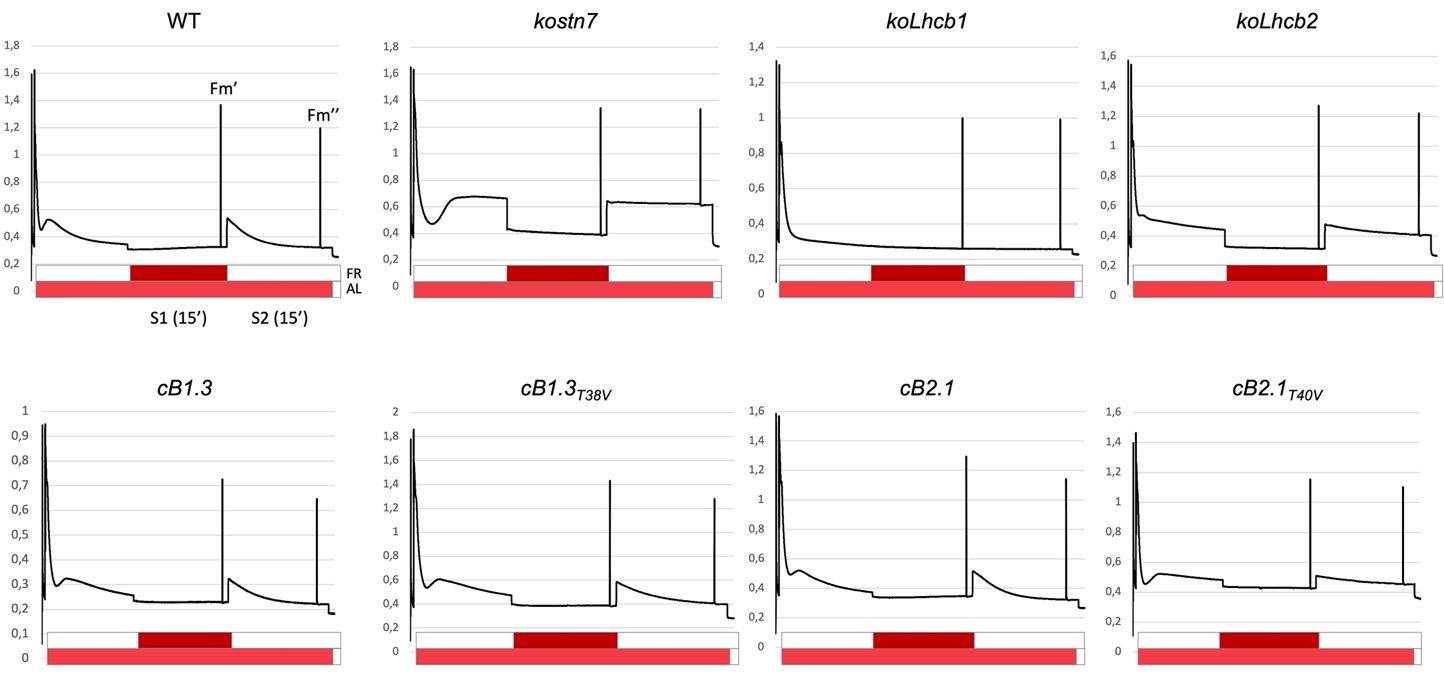
**

**Figure S.7: Fluorescence traces of all genotypes recorded during the PAM state transitions protocol.**

| **Species** | **Taxonomy** | **Sequence ID** | **Sequence** |
| --- | --- | --- | --- |
| *Arabidopsis thaliana* | Angiosperms  Dicots  *Brassicaceae* | A0A178WKX1 | MAASTMALSSPAFAGKAVKLSPAASEVLGSGRVTMRKTVAKPKGPSGSPWYGSDRVKYLGPFSGESPSYLTGEFPGDYGWDTAGLSADPETFARNRELEVIHSRWAMLGALGCVFPELLARNGVKFGEAVWFKAGSQIFSDGGLDYLGNPSLVHAQSILAIWATQVILMGAVEGYRVAGNGPLGEAEDLLYPGGSFDPLGLATDPEAFAELKVKELKNGRLAMFSMFGFFVQAIVTGKGPIENLADHLADPVNNNAWAFATNFVPGK |
| *Solanum lycopersicum* | Angiosperms  Dicots  *Solanaceae* | NP_001316911.1 | MAASTMALSSSTFAGKAVKLSPSSSEISGNGRITMRKTAAKPKPASSGSPWYGPDRVKYLGPFSGESPSYLTGEFPGDYGWDTAGLSADPETFAKNRELEVIHCRWAMLGALGCVFPELLARNGVKFGEAVWFKAGSQIFSEGGLDYLGNPSLVHAQSILAIWACQVVLMGAVEGYRIAGGPLGEVVDPLYPGGSFDPLGLAEDPEAFAELKVKEIKNGRLAMFSMFGFFVQAIVTGKGPLENLADHIADPVNNNAWAFATNFVPGK |
| Cucurbita pepo | Angiosperms  Dicots  *Cucurbitaceae* | XP_023513324.1 | MAASSMALSSPSLAGQAVKLSPSASDILGESRLTMRKTGGRPKPVSSGSPWYGPDRVKYLGPFSGEPPSY  LTGEFPGDYGWDTAGLSADPETFAKNRELEVIHSRWAMLGALGCVFPELLSRNGVKFGEAVWFKAGSQIFSEGGLDYLGNPSLVHAQSILAIWACQVVLMGAVEGYRIAGGPLGEITDPIYPGGSFDPLGLADDPEAFAELKVKELKNGRLAMFSMFGFFVQAIVTGKGPLENLADHLADPVNNNAWSYATNFVPGK |
| *Lemna gibba* | Angiosperms  Monocots  *Araceae* | M29334.1 | MAASMALSSPSLVGKAVKLAPAASEVFGEGRVSMRKTAGKPKPVSSGSPWYGPDRVKYLGPFSGEAPSYLTGEFAGDYGWDTAGLSADPETFAKNRELEVIHARWAMLGALGCVFPELLARNGVKFGEAVWFKAGSQIFSEGGLDYLGNPSLVHAQSILAIWATQVVLMGAVEGYRVAGGPLGEVVDPLYPGGSFDPLGLADDPEAFAELKVKEIKNGRLAMFSMFGFFVQAIVTGKGPLENLADHLADPVNNNAWAFATNFVPGK |
| *Triticum aestivum* | Angiosperms  Monocots  *Poaceae* | XP_044399748.1 | MAATTMSLSSPAFAGKAVKNLPSSSVFGEARVTMRKTAAKAKQVASGSPWYGSDRVLYLGPLSGEPPSYLTGEFPGDYGWDTAGLSADPETFAKNRELEVIHCRWAMLGALGCVFPELLARNGVKFGEAVWFKAGSQIFSEGGLDYLGNPSLVHAQSILAIWACQVVLMGAVEGYRIAGGPLGEIVDPLYPGGSFDPLGLADDPEAFAELKVKEIKNGRLAMFSMFGFFVQAIVTGKGPLENLADHLADPVNNNAWAFATNFVPGK |
| *Hordeum vulgare* | Angiosperms  Monocots  *Poaceae* | BAJ93064.1 | MAATTMSLSSPAFAGKAVKNLPSSSLFGEARVTMRKTAAKVKQVASGSPWYGSDRVLYLGPLSGEPPSYLTGEFPGDYGWDTAGLSADPETFAKNRELEVIHCRWAMLGALGCVFPELLARNGVKFGEAVWFKAGSQIFSEGGLDYLGNPSLVHAQSILAIWACQVVLMGAVEGYRIAGGPLGEIVDPLYPGGSFDPLGLADDPEAFAELKVKEIKNGRLAMFSMFGFFVQAIVTGKGPLENLADHLSDPVNNNAWAFATNFVPGK |
| *Oryza sativa* | Angiosperms  Monocots  *Poaceae* | NP_001393092.1 | MAAATMALSSPALAGKAAAKVFGEGRITMRKSAAKPKPAASGSPWYGADRVLYLGPLSGEPPSYLTGEFPGDYGWDTAGLSADPETFAKNRELEVIHSRWAMLGALGCVFPELLARNGVKFGEAVWFKAGSQIFSEGGLDYLGNPSLIHAQSILAIWAVQVVLMGAVEGYRIAGGPLGEVVDPLYPGGSFDPLGLADDPEAFAELKVKEIKNGRLAMFSMFGFFVQAIVTGKGPLENLADHLADPVNNNAWAYATNFVPGK |
| *Zea mays* | Angiosperms  Monocots  *Poaceae* | NP_001147639.2 | MAASTMAISSTAMAGTPIKVGSFGEGRITMRKTVGKPKVAASGSPWYGPDRVKYLGPFSGEPPSYLTGEFPGDYGWDTAGLSADPETFAKNRELEVIHSRWAMLGALGCVFPELLSRNGVKFGEAVWFKAGSQIFSEGGLDYLGNPSLIHAQSILAIWACQVVLMGAVEGYRIAGGPLGEVVDPLYPGGSFDPLGLADDPEAFAELKVKELKNGRLAMFSMFGFFVQAIVTGKGPLENLADHIADPVNNNAWAYATNFVPGN |
| *Glycine max* | Angiosperms  Dicots  *Fabaceae* | KAH1206782.1 | MAASTMALSSSSLAGQAIKLAPSTPQLGVGRVSMRKTASKTVSSGSPWYGPDRVKYLGPFSGEPPSYLTGEFPGDYGWDTAGLSADPETFAKNRELEVIHSRWAMLGALGCVFPELLARNGVKFGEAVWFKAGSQIFSEGGLDYLGNPSLIHAQSILAIWATQVILMGAVEGYRIAGGPLGEVTDPIYPGGSFDPLGLADDPEAFAELKVKELKNGRLAMFSMFGFFVQAIVTGKGPLENLADHLADPVNNNAWAYATNFVPGKNLKVQTKKLL |
| *Zostera marina* | Angiosperms  Monocots  *Zosteraceae* | KMZ74751.1 | MASSMAALSSPSLAGKAVKLAQEPNAIFGVGRITMRRNTVKKSVSSGSPWYGSDRVKYLGPFSGESPSYLTGEFPGDYGWDTAGLSADPETFSKNRELEVIHARWAMLGTLGCVFPELLSRNGVKFGEAVWFKAGSQIFSDGGLDYLGNSSLVHAQSILAIWATQVILMGAIEGYRVAGGPLGEIEDPLYPGGSFDPLNLAEDPEAFAEL |
| Sphagnum fallax | Briophyta  *Sphagnaceae* | KAH8957471.1 | MAAATACASSATFAGQTVLKQASELASKVGSSESRLQMRGSKAKASSGSIWYGADRPKYLGPFSGETPSYLTGEFAGDYGWDTAGLSADPETFARNRELEVIHARWAMLGALGCVTPELLAKNGTKFGEAVWFKAGSQIFADGGLDYLGNPSLVHAQSILAIWASQVVLMGAVEGYRVAGGPLGDVSDPIYPGGSFDPLGLADDPDTFSELKVKELKNGRLAMFSMFGFFVQAIVTGKGPLENLSDHLADPVTNNAWAYATNFTPGS |
| Picea glauca | Gymnosperms  *Pinaceae* | BT114202.1_mRNA | MASCGIGSRCAFTGGQLSSVKPQNNQLLGVGGGHGEARLTMRKATGKKSVAASSDSPWYGPDRVLYLGPFSGEPPSYLTGEFPGDYGWDTAGLSADPETFAKNRELEVIHSRWAMLGALGCVFPELLARNGVKFGEAVWFKAGAQIFSEGGLDYLGSPNLIHAQSILAIWACQVILMGAIEGYRVAGGPLGEVTDPIYPGGNFDPLGLADDPDAFAELKVKELKNGRLAMFSMFGFFVQAIVTDKGPIENLADHLADPVNNNAWAYATNFVPGK |
| *Diphasiastrum complanatum* | Lycophites  *Lycopodiaceae* | KAJ7566132.1 | MAAMAATALASPALVGQTSLKPQNEFLAKTGGSGQSRITMRKTFSKVASVSPWYGPDRVKYLGPFSGETPSYLTGEFPGDYGWDTAGLSADPETFAKNRELEVIHCRWAMLGALGCIFPELLSKNGVKFGEAVWFKAGAQIFQEGGLDYLGNPSLIHAQSILAIWATQVILMGAVEGYRVAGGPLGEVTDPIYPGGSFDPLGLADDPEAFAELKVKEIKNGRLAMFSMFGFFVQAIVTGKGPLENLSDHLADPVANNAWAYATNFVPGS |
| *Adiantum capillus-veneris* | Polypodiophyta  *Pteridaceae* | KAI5067263.1 | MAAAALSSSTFAGQAVYKPQSELSSKVGNVGEARVSMRKTVSKSSDSPWYGPDRVKYLGPFSGEAPSYLKGEFPGDYGWDTAGLSADPETFAKNRELEVIHARWAMLGALGCVTPELLAKNGVKFGEAVWFKAGSQIFAEGGLDYLGNPSLVHAQSILAIWACQVILMGAVEGYRVAGGPLGEVEDPIYPGGSFDPLGLADDPEAFAELKVKELKNGRLAMFSMFGFFVQAIVTGKGPIENLSDHLADPAINNAWAYATNFTPGK |
| *Physcomitrium patens* | Briophyta  *Funariaceae* | XP_024377170.1 | MATAATAMNSTVLAGQALLKPVSELSRKVNAGEARVTMRKTVSKSSGSDSIWYGADRPKFLGPFSGETPSYLNGEFAGDYGWDTAGLSSDPETFARNRELEVIHARWAMLGALGCLTPELLAKSGVKFGEAVWFKAGAQIFSEGGLDYLGNPSLVHAQSILAIWACQVILMGAVEGYRVAGGPLGDVTDPIYPGGSFDPLGLADDPDTFAELKVKEIKNGRLAMFSMFGFFVQAIVTGKGPLENLNDHLADPVANNAWAYATNFVPGN |
| *Marchantia polymorpha ruderalis* | Marchantiophyta  *Marchantiaceae* | OAE29815.1 | MASATVCASSTFAGQALGASSNALAAKVNVGEARVVMRKTVKSTPTSIWYGEDRPKYLGPFSGATPSYLTGEFPGDYGWDTAGLSADPETFAKNRELEVIHARWAMLGALGCVFPELLSKNGVSFGEAVWFKAGSQIFAEGGLDYLGNSSLVHAQSILAIWACQVVLMGAIEGYRVAGGPLGEVSDPIYPGGQFDPLNLAEDPDTFAELKVKELKNGRLAMFSMFGFFVQAIVTGKGPIENLSDHLADPVANNAWAYATNFTPGN |
| *Pteris vittata* | Polypodiophyta  *Pteridaceae* | HM179992.1 | MAASTASLSSATFSGQQLKSVNELSRKVGAGEARVQMMAPKKASTSGSIWYGADRPLYLGPFSGSPPSYLTGEFPGDYGWDTAGLSADPETFAKNRELELIHARWAMLGALGCVTPELLAKNGVKFGEAVWFKAGSQIFAEGGLDYLGNPSLVHAQSILAIWAFQVVLMGAVEGYRVAGGPLGEVEDPLYPGGSFDPLGLADDPEAFAELKVKELKNGRLAMFSMFGFFVQAIVTGKGPIENLSDHLADPTVNNAWAYATNFTPGK |

**Supplementary table 2: list of amino acid sequences of Lhcb1.3 orthologs of species from different taxonomic/phylogenetic groups used for multiple sequence analysis.**

| **Species** | **Taxonomic group** | **Sequence ID** | **Sequence** |
| --- | --- | --- | --- |
| *Arabidopsis thaliana* | Angiosperms  Dicots  *Brassicaceae* | AAD28769.1 | MATSAIQQSSFAGQTALKPSNELLRKVGVSGGGRVTMRRTVKSTPQSIWYGPDRPKYLGPFSENTPSYLTGEYPGDYGWDTAGLSADPETFAKNRELEVIHSRWAMLGALGCTFPEILSKNGVKFGEAVWFKAGSQIFSEGGLDYLGNPNLIHAQSILAIWAVQVVLMGFIEGYRIGGGPLGEGLDPLYPGGAFDPLNLAEDPEAFSELKVKELKNGRLAMFSMFGFFVQAIVTGKGPIENLFDHLADPVANNAWSYATNFVPGN |
| *Solanum lycopersicum* | Angiosperms  Dicots  *Solanaceae* | NP_001295874.1 | MATSAIQHSAFAGQTALKSQNEFIRKIGSFEGGRVTMRRTVKSAPQSIWYGEDRPKYLGPFSEQTPSYLTGEFPGDYGWDTAGLSADPETFARNRELEVIHCRWAMLGALGCVFPEILSKNGVTFGEAVWFKAGSQIFSEGGLDYLGNPNLIHAQSILAIWASQVVLMGFVEGYRVGGGPLGEGLDKIYPGGAFDPLGLADDPEAFAELKVKEIKNGRLAMFSMFGFFVQAIVTGKGPIENLSDHIADPVANNAWAYATNFVPGK |
| Cucurbita pepo | Angiosperms  Dicots  *Cucurbitaceae* | XP_023529768.1 | MATSAIQQSAFAGQTALKQSNELVRKVGAFGGSRFTMRRTVKSAPQSIWYGPDRPKYLGPFSEQTPSYLTGEFPGDYGWDTAGLSADPETFAKNRELEVIHSRWAMLGALGCVFPEVLAKNGVKFGESVWFKAGSQIFSEGGLDYLGNPNLVHAQSILAIWACQVVLMGFVEGYRVGGGPLGEGLDPIYPGGAFDPLGLADDPDAFAELKVKELKNGRLAMFSMFGFFVQAIVTGKGPIENLFDHVADPVANNAWAYATNFVPGK |
| *Lemna gibba* | Angiosperms  Monocots  *Araceae* | P12328.1 | MAASAIQSSAFAGQTALKQRDELVRKVGVSDGRFSMRRTVKAVPQSIWYGADRPKFLGPFSEQTPSYLTGEFPGDYGWDTAGLSADPETFAKNRELEVIHSRWAMLGALGCIFPELLSKNGVQFGEAVWFKAGAQIFSEGGLDYLGNPNLVHAQSILAIWATQVVLMGLIEGYRVGGGPLGEGLDPLYPGGAFDPLGLADDPEAFAELKVKEIKNGRLAMFSMFGFFVQAIVTGKGPIENLADHIADPVANNAWAFATNFVPGK |
| *Triticum aestivum* | Angiosperms  Monocots  *Poaceae* | XP_044382372.1 | MAASALHQTTSFLGTAPRRDDLVRSVGDFGGRITMRRTVKSAPQSIWYGPDRPKYLGPFSEQTPSYLTGEFPGDYGWDTAGLSADPETFAKNRELEVIHSRWAMLGALGCVFPEILSKNGIKFGEAVWFKAGAQIFSEGGLDYLGNPNLVHAQSILAIWAVQVVLMGFIEGYRVGGGPLGEGLDIIYPGGAFDPLGLADDPDTAAELKVKELKNGRLAMFSMFGFFVQAIVTGKGPVENLFDHVADPVNNNAWAFATNFAPGS |
| *Hordeum vulgare* | Angiosperms  Monocots  *Poaceae* | XP_044950450.1 | MGWRGRVAPRIHQLRPAHLSRRIKIHLAHRTSPPKHTHTHTILQLISLSSQAAPATSEMAASALHQTTSFLGTAPRRDDLVRSVGDFGGRITMRRTVKSAPQSIWYGPDRPKYLGPFSEQTPSYLTGEFPGDYGWDTAGLSADPETFAKNRELEVIHSRWAMLGALGCVFPEILSKNGIKFGEAVWFKAGAQIFSEGGLDYLGNPNLVHAQSILAIWACQVVLMGFIEGYRVGGGPLGEGLDIIYPGGAFDPLGLADDPDTAAELKVKELKNGRLAMFSMFGFFVQAIVTGKGPVENLFDHIADPVNNNAWAFATNFAPGS |
| *Oryza sativa* | Angiosperms  Monocots  *Poaceae* | ABF97414.1 | MAASALHQTTSFLGTAPRRDELVRRVGDSGGRITMRRTVKSAPQSIWYGPDRPKYLGPFSEQTPSYLTGEFPGDYGWDTAGLSADPETFARNRELEVIHSRWAMLGALGCVFPEILSKNGVKFGEAVWFKAGAQIFSEGGLDYLGNPNLVHAQSILAIWAVQVVLMGFVEGYRVGGGPLGEGLDKVYPGGAFDPLGLADDPDTFAELKVKELKNGRLAMFSMFGFFVQAIVTGKGPIENLFDHVADPVANNAWAYATNFVPGK |
| *Zea mays* | Angiosperms  Monocots  *Poaceae* | ACG30954.1 | MAASALHQTTSFLGQALVARAAGVDAGDRITMRRTVKSVPQSIWYGPDRPKYLGPFSEQTPSYLTGEFPGDYGWDTAGLSADPETFARNRELEVIHSRWAMLGALGCVFPEILAKNGVKFGEAVWFKAGAQIFSEGGLDYLGNPNLVHAQSILAIWACQVVLMGFVEGYRVGGGPLGEGLDKVYPGGAFDPLGLADDPDTAAELKVKELKNGRLAMFSMFGFFVQAIVTGKGPIENLFDHVADPVANNAWAYATNFVPGN |
| *Glycine max* | Angiosperms  Dicots  *Fabaceae* | AAL29886.1 | MATSAIQQSAFAGQTALKQLNELVRKTGGAGKGRTTMRRTVKSAPQSIWYGPDRPKYLGPFSEQIPSYLTGEFPGDYGWDTAGLSADPETFARNRELEVIHSRWAMLGALGCTFPEILEKNGVKFGEAVWFKAGSQIFSEGGLDYLGNPNLIHAQSILAIWAVQVVLMGFVEGYRVGGGPLGEGLDPIYPGGAFDPLGLADDPDAFAELKVKELKNGRLAMFSMFGFFVQAIVTGKGPIQNLYDHVADPVANNAWAYATNFVPGQ |
| *Zostera marina* | Angiosperms  Monocots  *Zosteraceae* | KMZ73176.1 | MATSAIHQSVFVGQTTLKQQNELVRKVGVFESRISMRRTVKSTPDSVWYGADRPKYLGPFSEQTPSYLTGEFPGDYGWDTAGLSADPETFAKNRELEVIHCRWAMLGALGCVFPELLSKNGVKFGEAVWFKAGSQIFSDGGLDYLGNPNLVHAQSILAIWACQVVLMGFIEGYRVGGGPLGDGLDPLYPGGSFDPLGLADDPEAFTELKVKEVKNGRLAMFSMFGYFVQAIVTGKGPIENLYDHLADPVANNAWAFATNFSPAN |
| Sphagnum fallax | Briophyta  *Sphagnaceae* | KAH8950389.1 | MATASAISSSTFAGQQVLKPQSELSRKVGNVEARVSMRRTLKSTPESIWYGPDRPKFLGPFSEATPSYLTGEFPGDYGWDTAGLSADPESFAKNRELEVIHSRWAMLGTLGMVFPELLAKNGVKFGEPIWFKAGSQIFSEGGLDYLGNPSLIHAQSILAIWATQVVLMGAIEGYRVGGGPLGEGLDPIYPGGQFDPLGLADDPDTFAELKVKELKNGRLAMFSAFGFFVQAIVTGKGPLENLTDHLADPVANNAWAYATKFTPGA |
| Picea glauca | Gymnosperms  *Pinaceae* | AAF97781.1 | MATASAIQISSLAGQTLLRPQQNELVSKMGASQARITMRRTVRSAPESIWYGPDRPKYLGPFSEQTPSYLTGEFPGDYGWDTAGLSADPETFAKNRELEVIHSRWAMLGALGCVFPELLAKTGVKFGEAVWFKAGRRYSRREASTTWGTPT |
| *Diphasiastrum complanatum* | Lycophites  *Lycopodiaceae* | KAJ7545057.1 | MAAITSASAISSPSFAGQTILKPQSELARKVGNVESRITMRRTVKSTPESIWYGPDRPKYLGPFSEQTPAYLTGEFPGDYGWDTAGLSADPETFAKNRELEVIHARWAMLGALGIVTPELLSKNGVKFGEAVWFKAGAQIFQEGGLDYLGNPNLIHAQSILAIWATQVVLMGFVEGYRVGGGPLGEGLDPIYPGGAFDPLGLADDPDTFAELKVKEIKNGRLAMFSVFGFFVQAIVTGKGPIENLTDHLADPATNNAWAYATNFVPGS |
| *Adiantum capillus-veneris* | Polypodiophyta  *Pteridaceae* | KAI5064311.1 | MATMTSASAISSSTFAGQTALKSQSELSKRVGNVDARVTMRRTVKSTPESIWYGPDRPKYLGPFSEATPSYLTGEFPGDYGWDTAGLSADPETFSKNRELEVIHARWAMLGALGCVTPELLAKNGVKFGEAVWFKAGSQIFADGGLDYLGNPSLVHAQSILAIWACQVVLMGAVEGYRVGGGPLGEGLDPVYPGGAFDPLGLADDPDSFAELKVKEIKNGRLAMFSMFGFFVQAIVTGKGPIENLSDHLADPTVNNAWAYATNFTPGK |
| *Physcomitrium patens* | Briophyta  *Funariaceae* | XP_024359073.1 | MAAATAMHSTTLAGQSLLKPVNELSRKVGSSEARVTMRRTVKSTSDSIWYGADRPKYLGPFSGETPSYLTGEFAGDYGWDTAGLSSDPETFARNRELEVIHARWAMLGALGCLTPELLAKSGVKFGEAVWFKAGAQIFSEGGLDYLGNPSLVHAQSILAIWASQVVLMGAVEGYRVAGGPLGEITDPIYPGGSFDPLGLADDPDTFAELKVKEIKNGRLAMFSMFGFFVQAIVTGKGPLENLNDHLADPVANNAWAYATNFVPGN |
| *Marchantia polymorpha ruderalis* | Marchantiophyta  *Marchantiaceae* | OAE29817.1 | MASATACASTVLAGQAIGASSNALAAKVNVGEARVVMRKTVKSTPTSIWYGEDRPKYLGPFSGATPSYLTGEFPGDYGWDTAGLSADPETFAKNRELEVIHARWAMLGALGCVFPELLSKNGVSFGEAVWFKAGSQIFAEGGLDYLGNSSLIHAQSILAIWACQVVLMGAIEGYRVAGGPLGEVSDPIYPGGQFDPLNLAEDPDTFAELKVKELKNGRLAMFSMFGFFVQAIVTGKGPIENLSDHLADPVANNAWAYATNFTPGN |
| *Pteris vittata* | Polypodiophyta  *Pteridaceae* | ADI88502.1 | MAASTASLSSATFSGQQLKSVNELSRKVGAGEARVQMMAPKKASTSGSIWYGADRPLYLGPFSGSPPSYLTGEFPGDYGWDTAGLSADPETFAKNRELELIHARWAMLGALGCVTPELLAKNGVKFGEAVWFKAGSQIFAEGGLDYLGNPSLVHAQSILAIWAFQVVLMGAVEGYRVAGGPLGEVEDPLYPGGSFDPLGLADDPEAFAELKVKELKNGRLAMFSMFGFFVQAIVTGKGPIENLSDHLADPTVNNAWAYATNFTPGK |

**Supplementary table 2: list of amino acid sequences of Lhcb2.1 orthologs of species from different taxonomic/phylogenetic groups used for multiple sequence analysis.**

**
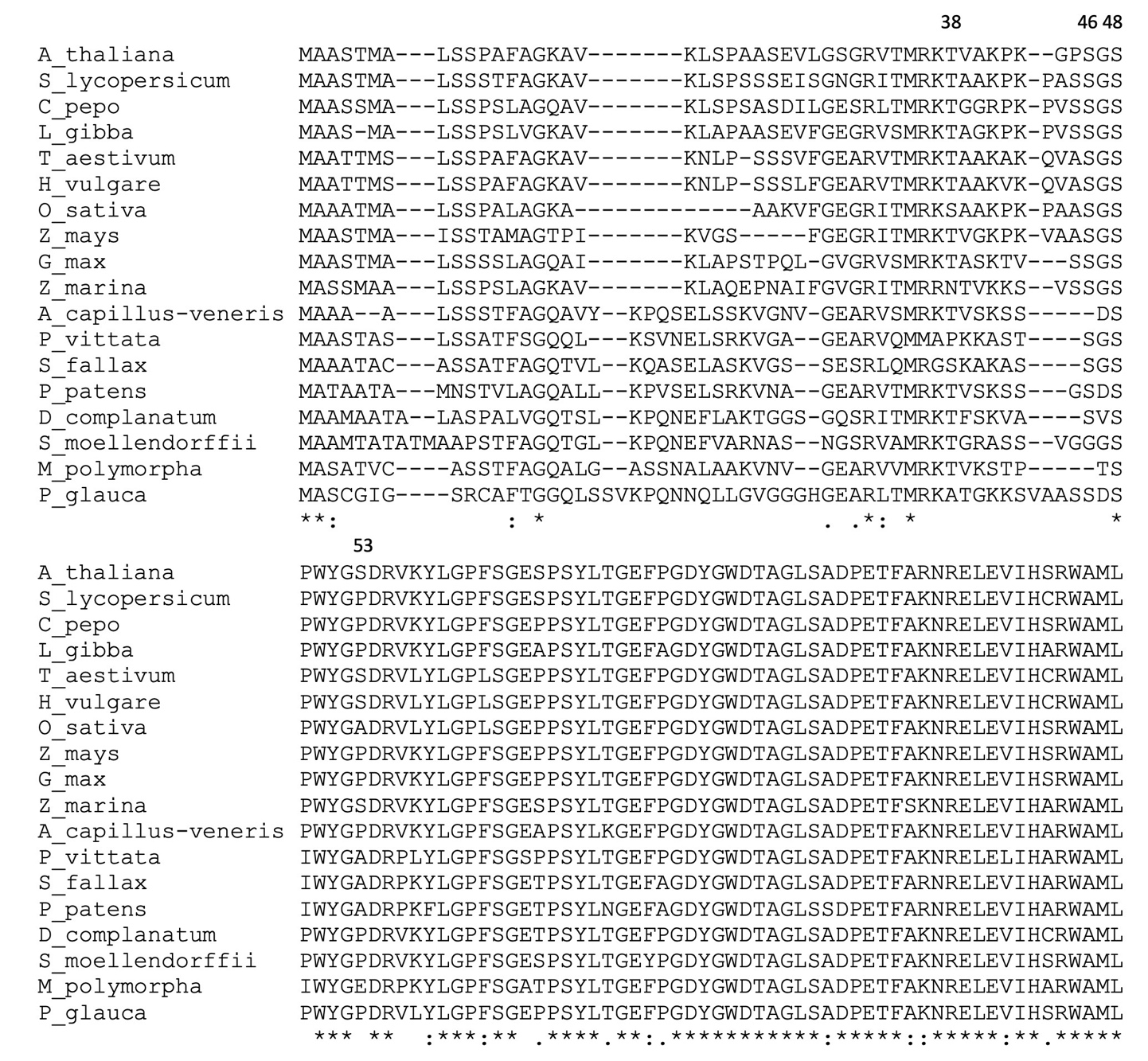
**

**Figure S.8: multiple sequence alignment of Lhcb1 protein isoforms of representative species from major taxonomic/phylogenetic groups.**

**
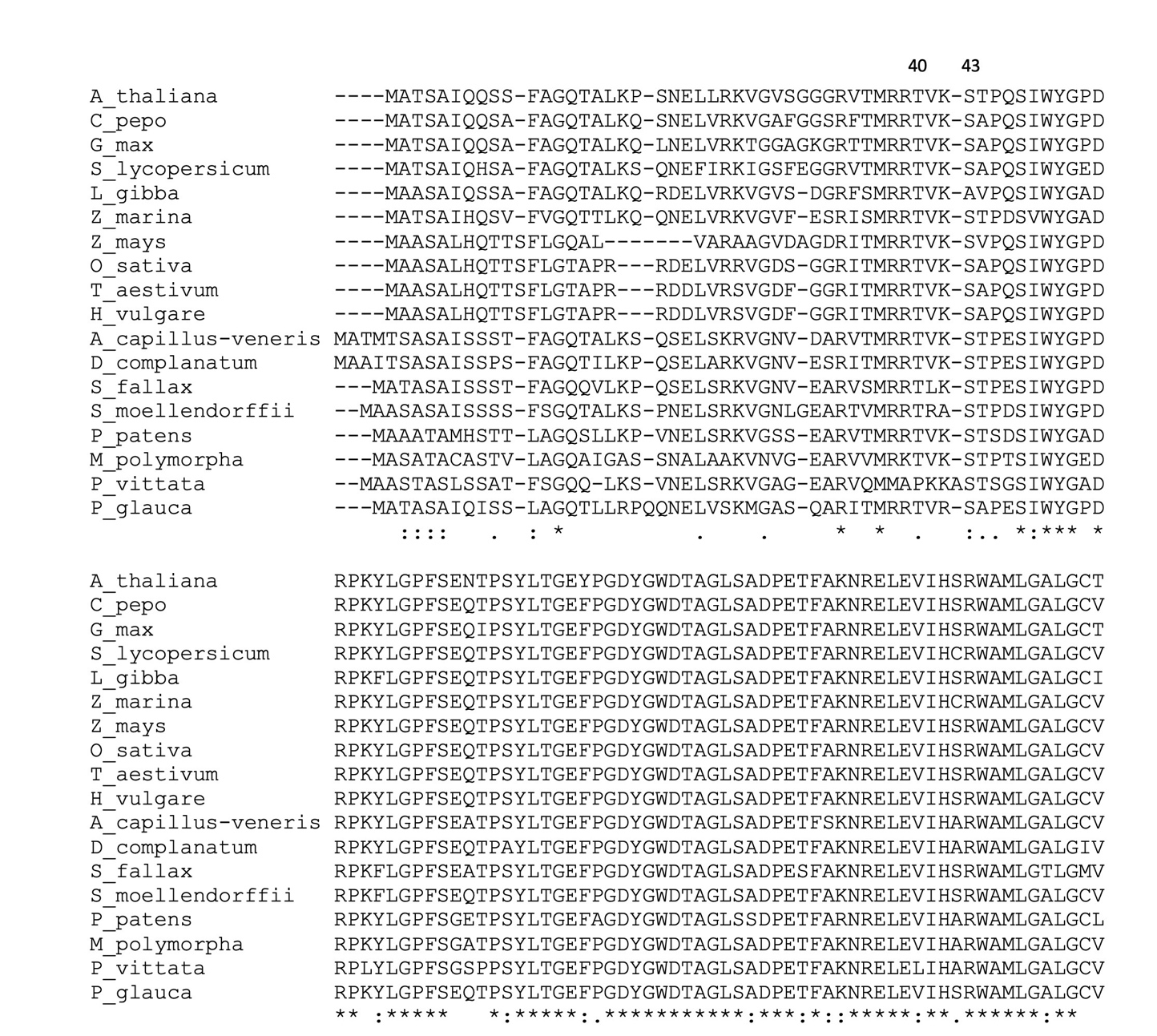
**

**Figure S.9: multiple sequence alignment of Lhcb2 protein isoforms of representative species from major taxonomic/phylogenetic groups.**
